# Downregulation of microRNA‐330‐5p induces manic‐like behaviors in REM sleep‐deprived rats by enhancing tyrosine hydroxylase expression

**DOI:** 10.1111/cns.14121

**Published:** 2023-02-16

**Authors:** Kang Hyun Leem, Sanga Kim, Hee Won Kim, Hae Jeong Park

**Affiliations:** ^1^ Department of Herbology, College of Korean Medicine Semyung University Jecheon Korea; ^2^ Department of Pharmacology, School of Medicine Kyung Hee University Seoul Korea; ^3^ Department of Medical Engineering, Graduate School Kyung Hee University Seoul Korea

**Keywords:** manic‐like behavior, miR‐330‐5p, prefrontal cortex, rapid eye movement sleep deprivation, tyrosine hydroxylase

## Abstract

**Aim:**

In our pilot study, we found an increase in tyrosine hydroxylase (Th) mRNA expression in the prefrontal cortex of 72‐h REM sleep‐deprived (SD) rats, a mania model. Additionally, the expression levels of miR‐325‐3p, miR‐326‐3p, and miR‐330‐5p, the predicted target miRNAs on TH, were significantly decreased. Based on these results, in this study, we investigated whether miRNA‐325‐3p, miR‐326‐3p, and miR‐330‐5p modulate TH and manic‐like behaviors in SD rats.

**Methods:**

Manic‐like behaviors were assessed using the open field test (OFT) and elevated plus‐maze (EPM) test. The direct binding activity of miRNAs to the 3′‐untranslated region (3′‐UTR) of the *Th* gene was measured in HEK‐293 cells using a luciferase reporter system. We also examined mRNA and protein expression of TH after intracerebroventricular (ICV) injection of miR‐330‐5p agomir to SD rats, along with manic‐like behaviors.

**Results:**

We observed an upregulation in mRNA and protein expression of TH and downregulation in miRNA‐325‐3p, miR‐326‐3p, and miR‐330‐5p expressions in the prefrontal cortex of SD rats, together with increased manic‐like behaviors. The luciferase reporter assay showed that miR‐330‐5p could repress TH expression through direct binding to its target site in the 3′‐UTR of Th, whereas miR‐326‐3p and miR‐330‐5p could not. In addition, ICV injection of miR‐330‐5p agomir alleviated the increase in TH expression in the prefrontal cortex of SD rats and manic‐like behaviors.

**Conclusions:**

TH expression regulation through miR‐330‐5p may be implicated in the pathophysiology of mania in SD rats.

## Graphical abstract

In sleep‐deprived (SD) rats, manic‐like behaviors are involved in an increase in TH expression. Sleep deprivation decreases the expression of miR‐330‐5p, a target miRNA on TH, in the prefrontal cortex. Sleep deprivation directly attenuates miR‐330‐5p expression, which in turn, increases TH expression in the prefrontal cortex. The regulation of TH through miR‐330‐5p may be implicated in the pathophysiology of mania.

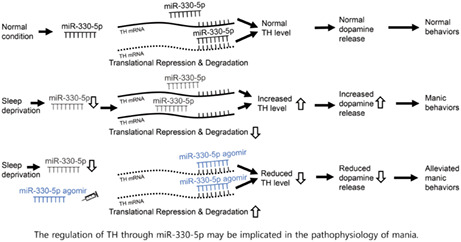

## 1 INTRODUCTION

Bipolar disorder (BPD) is a common psychiatric disorder characterized by symptoms ranging from hyperactive mania to depression, with periods of euthymia, in between. Sleep disturbance has been observed in various neurological and psychiatric disorders.^1^, ^2^, ^3^, ^4^ In patients with BPD, sleep loss is suggested as a trigger factor of manic episodes.^2^, ^3^ In addition, during the manic phase, patients with BPD showed decreased sleep, together with elevated mood such as increased energy, hyperactivity, and impulsivity.^5^, ^6^ A number of animal studies have proposed rodents subjected to sleep deprivation as a mania model.^7^, ^8^, ^9^ Indeed, sleep‐deprived (SD) rodents exhibit manic‐like behaviors, including hyperlocomotion, irritability, impulsivity, and aggressiveness, as observed during the manic phase in patients with BPD.^7^, ^8^, ^9^, ^10^

MicroRNAs (miRNAs) are highly conserved small non‐coding RNAs across species, with ~22 nucleotides in length. In animal cells, miRNAs bind to complementary sequences at the 3′‐untranslated region (3′‐UTR) of their target mRNAs, promoting translational repression and mRNA degradation by deadenylation and decapping.^11^, ^12^ Particularly, miRNAs interact with the 3′‐UTR of target mRNAs via Watson‐Crick base pairing of their 5′ seed region (nucleotides 2–8).^11^, ^12^ Aberrant miRNA expression has been associated with BPD.^13^, ^14^, ^15^, ^16^, ^17^, ^18^ Some miRNAs such as miR‐185‐5p, miR‐142‐3p, miR‐7‐5p, and miR‐107 have been shown to be associated with the mania of BPD through analysis of miRNA expressions in the peripheral blood of patients with BPD.^14^, ^15^, ^16^, ^17^ Squassina et al.^18^ measured miRNA expression in lymphoblastoid cell lines and postmortem brains of patients with BPD who died by suicide and found alterations in miR‐4286 expression. Although studies have reported the involvement of some miRNAs in BPD, there is a lack of knowledge regarding BPD‐associated miRNAs and the effects of miRNAs on their target mRNAs in BPD.

In our pilot study, we analyzed alterations in mRNA and miRNA expressions in the prefrontal cortex of 72‐h rapid eye movement (REM) SD rats using next‐generation sequencing (Tables S1–S8 and Figure S1). RNA sequencing (RNA‐seq) results showed that 55 significant pathways were altered by sleep deprivation (Table S5). A number of neurotransmitters have been implicated in the pathology of mania, including dopamine, serotonin, norepinephrine, and γ‐aminobutyric acid. Among the neurotransmitter synapse pathways, we found that the dopaminergic synapse pathway had the lowest *p*‐value (false discovery rate‐corrected *p* = 0.001) (Tables S5 and S6). Previous studies have also consistently reported dysregulation of dopaminergic neurotransmission in the mania of BPD.^19^, ^20^, ^21^, ^22^, ^23^ In particular, we focused on tyrosine hydroxylase (Th) showing the most potent alteration by sleep deprivation among the detected dopamine synapse genes (fold change = 7.814). TH is responsible for catalyzing the conversion of l‐tyrosine to l‐3,4‐dihydroxyphenylalanine (l‐DOPA), a precursor of dopamine. This is the rate‐limiting step in dopamine synthesis, and an increase in dopamine release induces an elevation in TH levels.^19^ Thus, the increase of Th expression in the prefrontal cortex of SD rats may be caused by the increased release of dopamine, which may be involved in manic‐like behaviors. Moreover, miRNA‐seq revealed 74 differentially expressed miRNAs (DEmiRNAs) in the prefrontal cortex of SD rats (Table S8). Based on the 74 DEmiRNAs, we analyzed the predicted target miRNAs on TH using the TargetScan v8.0 database (https://www.targetscan.org/vert_80/). Because miRNAs promote mRNA degradation and translation repression by binding to their target sites in the 3′‐UTR of genes,^11^ we selected the decreased miRNAs, considering the increased Th mRNA expression in SD rats. Finally, miR‐325‐3p, miR‐326‐3p, and miR‐330‐5p were selected as predicted target miRNAs on TH (Table S9). In this study, we investigated whether miR‐325‐3p, miR‐326‐3p, and miR‐330‐5p could modulate TH expression and affect manic‐like behaviors in SD rats.

## 2 MATERIALS AND METHODS

### 2.1 Animals and sleep deprivation

Male Sprague–Dawley rats (8 weeks old) were purchased from Central Lab. Animal Inc. The rats were maintained under controlled 12:12 light–dark, temperature (22 ± 2°C), and humidity (50 ± 10%) conditions with food and water *ad libitum*. All rats underwent an acclimatization period of 1 week before starting experimental procedures. All animal experiments were conducted in accordance with the Animal Care and Use Guidelines issued by Kyung Hee University and approved by the Animal Care and Use Committee of Kyung Hee University [KHUASP(SE)‐17‐042 and KHSASP‐20‐275].

For REM sleep deprivation, the modified multiple platform method was applied.^10^, ^24^ In brief, cages with six small platforms (6 cm diameter) were prepared and filled with water up to approximately 2 cm below the surface of the platform. Rats were placed on these small platforms (*n* = 3 per cage). When the rats entered REM sleep, they touched the water due to diminished muscle tone and were awakened. For control rats, cages with six large platforms (15 cm diameter) were prepared and filled with water (*n* = 3 per chamber). The platforms were large enough for the rats to sleep undisturbed but not for walking around freely because of the surrounding water. All rats were put on the platforms at the same time and remained in the cages for 72 h (from 10:00 a.m. on the first day to 10:00 a.m. on the fourth day). Food and water were provided ad libitum.

In the experiment for comparison between SD and control, a total of 24 rats were randomly assigned to control and SD groups (*n* = 12 per group). All rats were sacrificed after the open‐field test (OFT) and elevated plus‐maze (EPM) test, and the prefrontal cortex from each rat were dissected out. The prefrontal cortex of the left and right sides was separately stored at −80°C until analysis (a total of 24 samples per group). Four samples for RNA‐seq and miRNA‐seq, six samples for mRNA and miRNA isolation, and six samples for protein isolation were used randomly, regardless of left or right sides.

### 2.2 Intracerebroventricular injection

To examine the effect of miR‐330‐5p in SD rats, miR‐330‐5p or negative control (NC; scrambled oligonucleotides) agomirs (GenePharma) were injected into the rats via intracerebroventricular (ICV) injection. After anesthetization with avertin (250 mg/kg; i.p.), rats were mounted on a stereotaxic apparatus. The miR‐330‐5p and NC agomirs (0.25 nmol/rat) complexed with Invivofectamine 3.0 (Thermo Fisher Scientific) were injected with a volume of 5 μL into the right lateral ventricle of rats at the rate of 1 μL/min. The stereotaxic coordinates for ICV injection were as follows: −0.9 mm anterior, −1.8 mm lateral, and −3.8 mm relative to the dural surface from the bregma,^25^ according to the atlas of Paxinos and Watson.^26^ After recovery for 3 days, the rats were subjected to sleep deprivation.

### 2.3 Open‐field test

After 72 h of sleep deprivation, the locomotor activity of rats was measured using black open field plexiglass boxes (90 × 90 × 50 cm).^10^, ^27^ Each rat was placed in the center of the apparatus and allowed to explore the open field for 7 min while being recorded using a video camera. Locomotion was analyzed using ImageJ software (National Institutes of Health [NIH]), and the traveled distance for 5 min (00:00:30–00:05:30) was measured.

### 2.4 Elevated plus‐maze test

We next performed the EPM test using an EPM made of black wood.^10^ The EPM comprised two open arms (50 × 10 cm) and enclosed arms without a roof (50 × 10 × 30 cm) and was elevated to a height of 50 cm. After habituation in the testing room for at least 20 min, each rat was placed in the center of a cross maze, and their behaviors were recorded for 5 min using a video camera. The time spent and the number of entries into the open or closed arms were measured.

### 2.5 Cell culture and miRNA transfection

Rat adrenal gland PC12 cells were supplied by the Korean Cell Line Bank (KCLB; 21721). The cells were cultured in Dulbecco's Modified Eagle Medium supplemented with 10% fetal bovine serum and 100 U/mL penicillin/streptomycin. Cells were maintained in a humidified incubator at 37°C in an atmosphere containing 5% CO_2_. The cell culture medium was changed every 2 days.

To evaluate the effect of miRNAs on TH expression, mimics, and inhibitors of miR‐325‐3p, miR‐326‐3p, miR‐330‐5p, and NC (GenePharma) (100 nM) were transfected into PC12 cells using Lipofectamine RNAiMAX (Thermo Fisher Scientific). After 2 h of transfection, PC12 cells were treated with dexamethasone (Dex) (5 or 50 μM; Sigma–Aldrich) for 48 h to increase TH levels.^28^, ^29^

### 2.6 Luciferase reporter assay

The direct binding activity of miRNAs to the 3′‐UTR of the Th gene was assessed using a luciferase reporter system. Oligonucleotide segments on the 3′‐UTR of the Th gene containing the predicted target sites of each miRNA were cloned into the pmirGLO dual‐luciferase reporter vector (Promega).

Human embryonic kidney (HEK)‐293 cells (ATCC, CRL‐1573) were seeded into 24‐well plates. Cells were cotransfected with the pmirGLO reporter construct and the pRL‐TK vector at a ratio of 50:1 using Lipofectamine LTX (Thermo Fisher Scientific). After 6 h, the medium was changed and miR‐325‐3p, miR‐326‐3p, miR‐330‐5p, and NC (GenePharma) mimics (100 nM) were transfected using Lipofectamine RNAiMAX (Thermo Fisher Scientific). After 48 h, the cells were lysed and luciferase activity was assessed using the Dual‐Luciferase Reporter Assay System (Promega). Firefly luciferase activity was normalized to Renilla luciferase activity.

### 2.7 Quantitative PCR

For analyzing mRNA expression levels, total RNA was extracted using the RNeasy Mini kit (Qiagen). cDNA was synthesized from total RNA using the 1st Strand cDNA Synthesis Kit (BioAssay Co.) and random hexamers. Quantitative PCR (qPCR) was performed using a Real‐Time PCR EvaGreen Kit (SolGent) and specific primers for each gene (Table S10). The housekeeping gene Ubc was used as an internal reference control. To confirm the amplification specificity of the PCR products, melting curve analysis was performed after the completion of PCR cycling.

miRNAs were isolated using the miRNeasy Mini Kit (Qiagen). miRNA‐specific cDNAs were synthesized using specific stem‐loop and forward primers for each miRNA (Table S10).^30^ qPCR was conducted using Real‐Time PCR Kit (SolGent), Prob #21 (Roche), and gene‐specific forward and universal reverse primers (Table S10). The expression of U87 was assessed as the internal control.

qPCR was performed using the StepOnePlus Real‐Time PCR System (Applied Biosystems Inc.). The relative expressions of mRNA and miRNA transcripts were calculated using the $2^{-\Delta \Delta C_{T}}$ method.^31^

### 2.8 Western blotting

Proteins were extracted using the RIPA buffer (Sigma–Aldrich). Equal amounts of proteins (50 μg) were electrophoresed on sodium dodecyl sulfate‐polyacrylamide gels and then transferred onto a nitrocellulose membrane (Amersham Biosciences). After blocking with 5% skim milk, the membranes were incubated with rabbit TH primary antibody (Cell Signaling Technology) at 4°C overnight, followed by incubation with anti‐rabbit IgG conjugated to horseradish peroxidase (GeneTex). The protein bands were visualized using an enhanced chemiluminescence substrate (Bio‐Rad Laboratories). Band intensities were quantified using the ImageJ software (NIH).

### 2.9 Statistical analysis

The results are expressed as means ± standard errors of the mean (SEMs). Statistical analyses were performed using IBM SPSS Statistics 23 (SPSS Inc.). All data followed a normal variable distribution, which was checked by the Shapiro–Wilk test, together with the homogeneity of variances verified by the Levene test. Differences between groups were evaluated using an unpaired *t*‐test or one‐way analysis of variance (ANOVA), followed by the LSD post‐hoc test. Statistical significance was set at *p* < 0.05.

## 3 RESULTS

### 3.1 Manic‐like behaviors in SD rats

**FIGURE 1:**
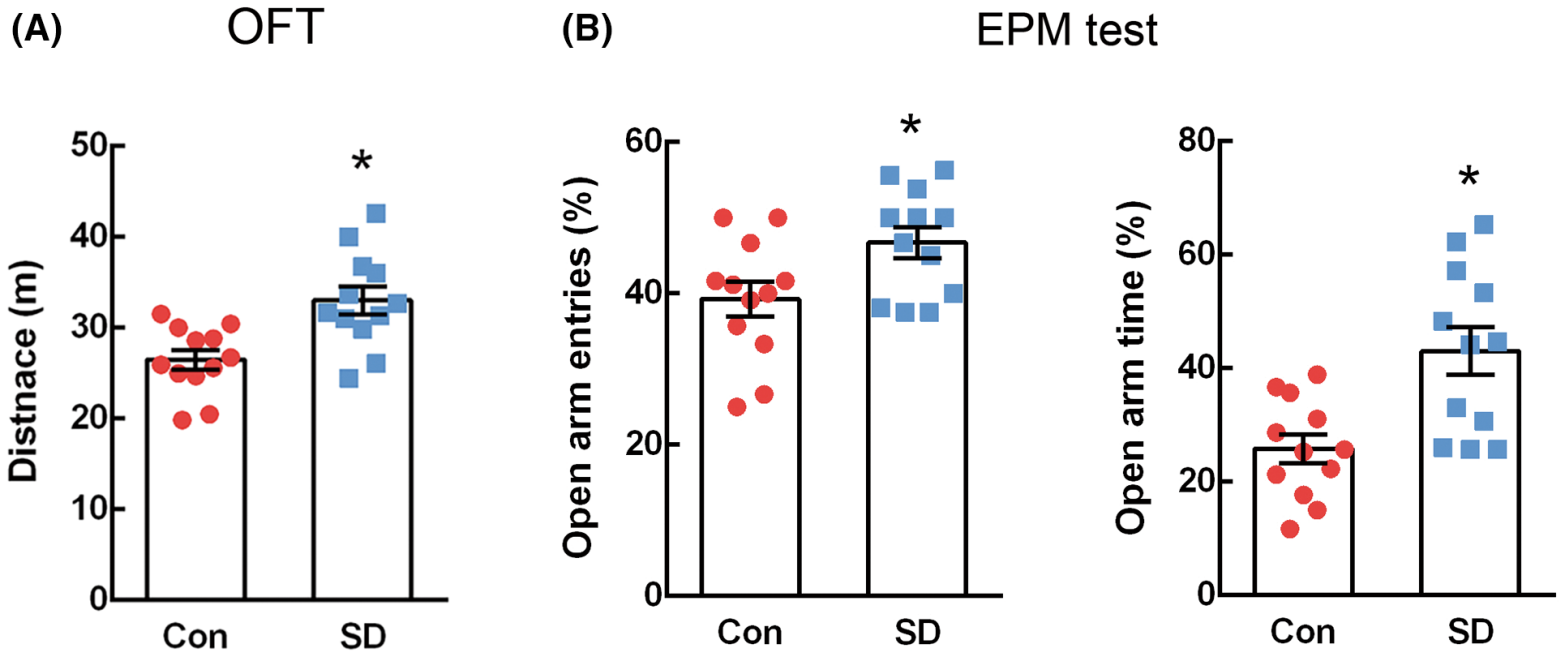
Manic‐like behaviors in rats subjected to sleep deprivation. Manic‐like behaviors in rats exposed to sleep deprivation for 72 h were measured using the open field test (A) and elevated plus maze (EPM) test (B). Hyperactive locomotion was indicated by an increase in total distance traveled in the open field test. Impulsive behavior was indicated by an increase in the percentage of open‐arm entries and time spent in the open arms. Data are presented as mean ± SEM (*n* = 12 per group). The difference between the sleep‐deprived and control groups was determined via an unpaired *t*‐test (*p < 0.05).

To examine whether SD induced hyperactive‐ and impulsive‐like behaviors, OFT and EPM tests were conducted in control and SD rats (*n* = 12 per group). The OFT showed increased locomotor activity in SD rats. As shown in Figure 1A, the total travel distance was significantly higher in the SD rats than in the control rats (*t* = −3.522, *p* = 0.002).

In the EPM test, increased frequency of open‐arm entries and increased time spent in open arms were considered signs of impulsive‐like behavior.^32^, ^33^ The number of open‐arm entries (*t* = −2.415, *p* = 0.024) and time spent in open arms (*t* = −3.496, *p* = 0.002) was higher in the SD rats compared to control rats (Figure 1B). These results indicate that SD induces manic‐like behaviors, such as hyperlocomotion and impulsivity, in rats.

### 3.2 Expressions of TH and predicted target miRNAs on TH in the prefrontal cortex of SD rats

**FIGURE 2:**
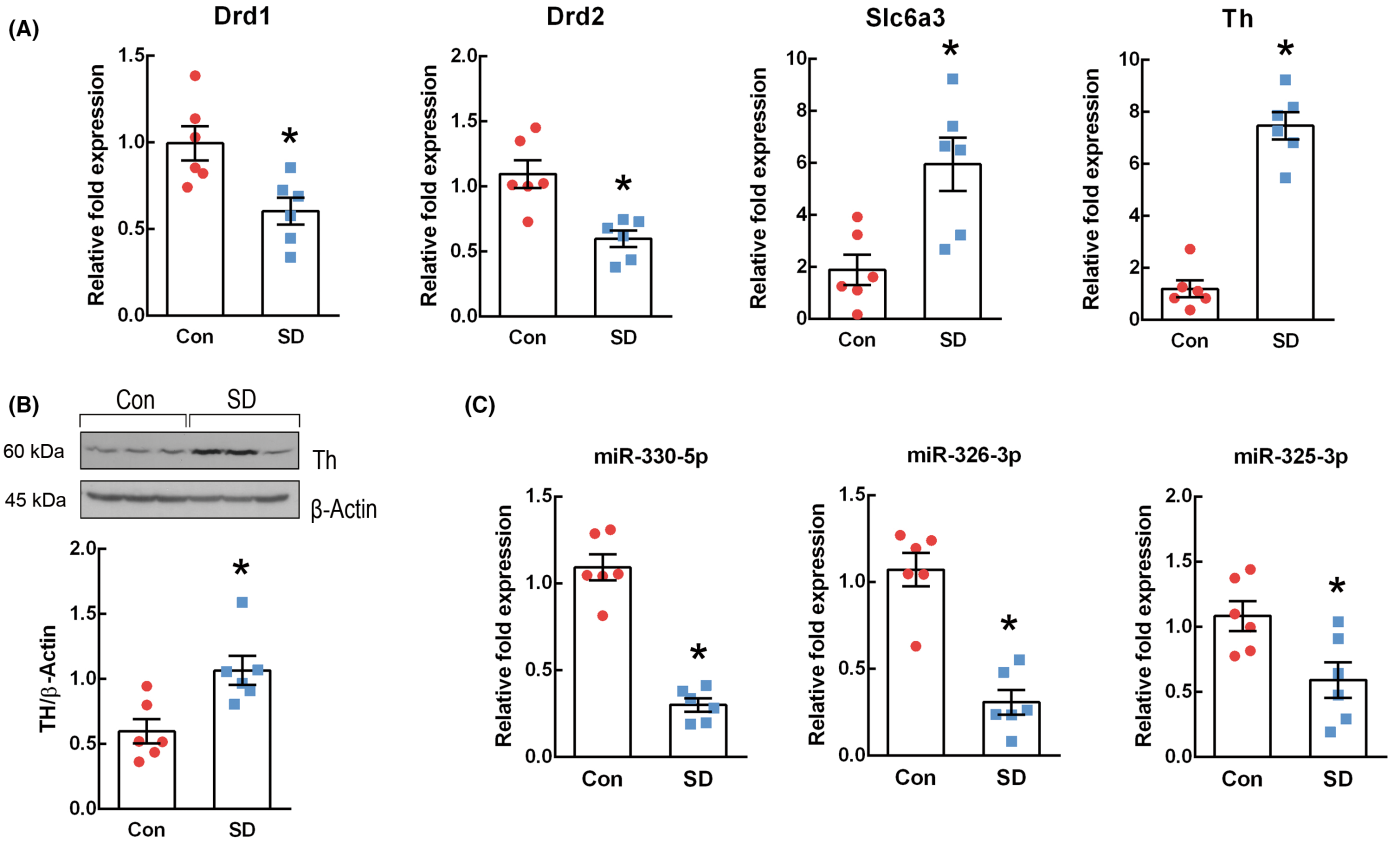
Expressions of tyrosine hydroxylase (TH) and predicted target microRNAs (miRNAs) on TH in sleep‐deprived (SD) rats. (A) Expressions of the dopaminergic synapse genes, including TH, were determined using quantitative PCR (qPCR) in the prefrontal cortex of control and SD rats (*n* = 6 per group). All samples were assayed in duplicate, and the mean of the duplicate values are represented. Gene expression levels were normalized against Ubc levels. (B) TH protein expression was assessed in the prefrontal cortex of rats by western blotting (*n* = 6 per group). β‐Actin was used as the internal reference control. (C) The expressions of the predicted target miRNAs on TH in the prefrontal cortex (*n* = 6 per group) were also detected using qPCR. The assay was performed in duplicate, and the mean of the duplicate values are represented. Gene expression levels were normalized against U87 levels. Results are shown as mean ± SEM. Differences between the SD and control groups were compared via unpaired *t*‐test (*p < 0.05).

In the prefrontal cortex of SD rats, we validated the altered expression of dopamine synapse genes observed in our pilot study using qPCR. As shown in Figure 2A, sleep deprivation decreased the mRNA expressions of dopamine receptor D1 (Drd1) (*t* = 3.127, *p* = 0.011) and Drd2 (*t* = 3.983, *p* = 0.003) and increased the mRNA expressions of solute carrier family 6 member 3 (Slc6a3) (*t* = −3.439, *p* = 0.006) and Th (*t* = −10.115, *p* < 0.001). We also examined TH protein expression (Figure 2B) and found that it was significantly elevated in the prefrontal cortex of SD rats compared to that in control rats (*t* = −3.233, *p* = 0.009).

The expressions of predicted target miRNAs on TH, miR‐330‐5p, miR‐326‐3p, and miR‐325‐3p, were also assessed in the prefrontal cortex of SD rats (Figure 2C). Sleep deprivation significantly decreased the expression levels of miR‐330‐5p (*t* = 9.434, *p* < 0.001), miR‐326‐3p (*t* = 6.367, *p* < 0.001), and miR‐325‐3p (*t* = 2.759, *p* = 0.020).

### 3.3 Effects of miR‐325‐3p, miR‐326‐3p, and miR‐330‐5p on TH mRNA and protein expression in Dex‐treated PC12 cells

**FIGURE 3:**
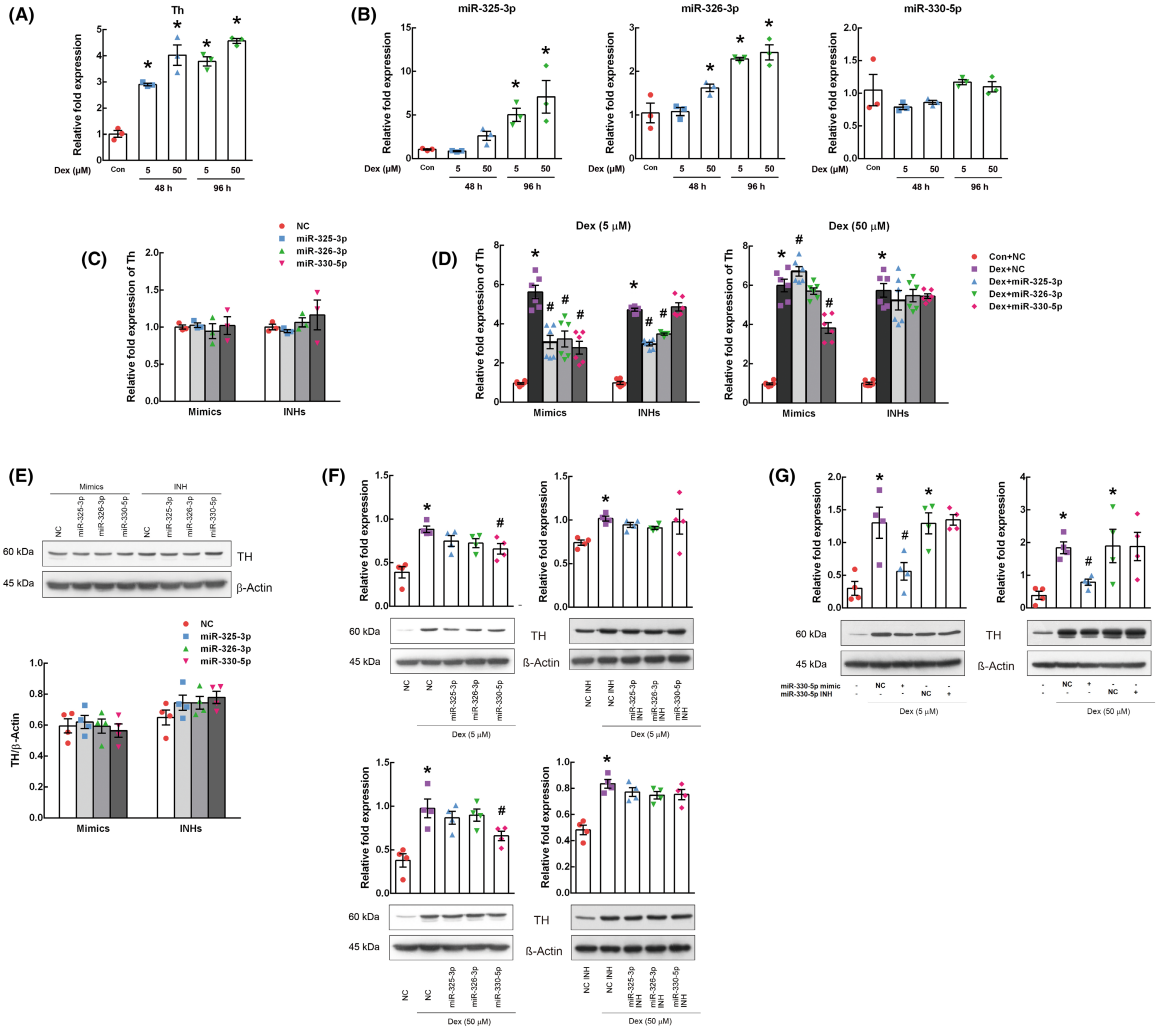
Effect of mimics and inhibitors (INHs) of miR‐325‐3p, miR‐326‐3p, and miR‐330‐5p on the expression of tyrosine hydroxylase (TH). TH mRNA and protein expressions were detected in dexamethasone (Dex)‐treated PC12 cells after transfection with miRNA mimics and INHs. Th mRNA expression was examined in PC12 cells treated with 5 or 50 μM Dex for 48–96 h (A), together with miRNAs (B). Next, the effects of miRNA mimics and INHs on TH mRNA expression were examined in cells treated without (C) and with Dex (5 or 50 μM) for 48 h (D). The effect of those on TH protein expression was also assessed under the same condition without (E) and with Dex (F, G). Expression levels of mRNAs and miRNAs were normalized against the levels of Ubc and U87, respectively. The protein expression level was normalized against β‐actin level. Independent experiments were repeated in triplicates. Data are presented as mean ± SEM. The difference between the groups was assessed by one‐way analysis of variance, followed by LSD post‐hoc test (*p < 0.05, compared to control or negative control [NC] mimic; ^#^ *p* < 0.05, compared to Dex with NC mimic).

To determine whether miR‐325‐3p, miR‐326‐3p, and miR‐330‐5p modulated TH, the mRNA and protein expression of TH were examined in Dex‐treated PC12 cells after transfection with miRNA mimics and inhibitors. First, we confirmed that Dex (5 and 50 μM) increased Th mRNA expression (*F*_4,10_ = 46.431, *p* < 0.001; Figure 3A). We also found that Dex increased the expression of miR‐325‐3p (*F*_4,10_ = 8.166, *p* = 0.003) and miR‐326‐3p (*F*_4,10_ = 21.736, *p* < 0.001) dose‐ and time‐dependently but not miR‐330‐5p (*F*_4,10_ = 1.951, *p* = 0.178; Figure 3B). These results indicate that Th mRNA expression could be elevated, despite the increase in miR‐325‐3p and miR‐326‐3p in Dex‐treated cells. miR‐325‐3p and miR‐326‐3p may not contribute to TH suppression or not enough.

Next, we observed that all mimics of miR‐325‐3p, miR‐336‐3p, and miR‐330‐5p repressed the increase in Th expression in 5 μM Dex‐treated cells (*F*_4,25_ = 27.258, *p* < 0.001 by one‐way ANOVA; *p* < 0.001 compared to Dex‐treated cells in post‐hoc test) (Figure 3D). However, in cells treated with 50 μM Dex, only the miR‐330‐5p mimic repressed Th mRNA expression (*F*_4,25_ = 100.680, *p* < 0.001 by One‐way ANOVA; *p* < 0.001, compared to Dex‐treated cells in post‐hoc test). In the cells transfected with miRNA inhibitors (Figure 3D) (by one‐way ANOVA, *F*_4,25_ = 189.49, *p* < 0.001 at 5 μM Dex and *F*_4,25_ = 41.032, *p* < 0.001 at 50 μM Dex), miR‐325‐3p and miR‐336‐3p inhibitors suppressed Th expression at 5 μM Dex, similar to their mimics (*p* < 0.001, compared to Dex‐treated cells in post‐hoc test), but not 50 μM. In contrast, the miR‐330‐5p inhibitor did not affect the Th level at either dose of Dex.

The modulatory effect of the miRNAs on TH was also examined at the protein level (by one‐way ANOVA, *F*_4,15_ = 11.999, *p* < 0.001 at 5 μM Dex and *F*_4,15_ = 9.524, *p* < 0.001 at 50 μM Dex). As shown in Figure 3F, miR‐325‐3p and miR‐326‐3p mimics did not suppress TH protein expression at any Dex dose. In contrast, the miR‐330‐5p mimic significantly suppressed the increase in TH protein expression at both 5 and 50 μM Dex (*p* = 0.012 and *p* = 0.012, respectively). In miRNA inhibitor‐transfected cells (by one‐way ANOVA, *F*_4,15_ = 2.449, *p* = 0.091 at 5 μM Dex and *F*_4,15_ = 15.838, *p* < 0.001 at 50 μM Dext), miRNA inhibitors did not affect TH expression in Dex‐treated cells at any doses (*p* > 0.05 in post‐hoc test). As shown in Figure 3G, we confirmed the effects of the miR‐330‐5p mimic and inhibitor, again (by one‐way ANOVA, *F*_4,15_ = 10.313, *p* < 0.001 at 5 μM Dex and *F*_4,15_ = 5.126, *p* = 0.008 at 50 μM Dex). The miR‐330‐5p mimic significantly decreased TH protein expression compared to Dex‐treated cells (*p* = 0.004 and *p* = 0.033 on 5 and 50 μM Dex, respectively). These results indicate that TH mRNA and protein expression is significantly suppressed by miR‐330‐5p but not by miR‐325‐3p and miR‐336‐3p.

### 3.4 Binding activity of miR‐325‐3p, miR‐326‐3p, and miR‐330‐5p on the 3′‐UTR of Th

**FIGURE 4:**
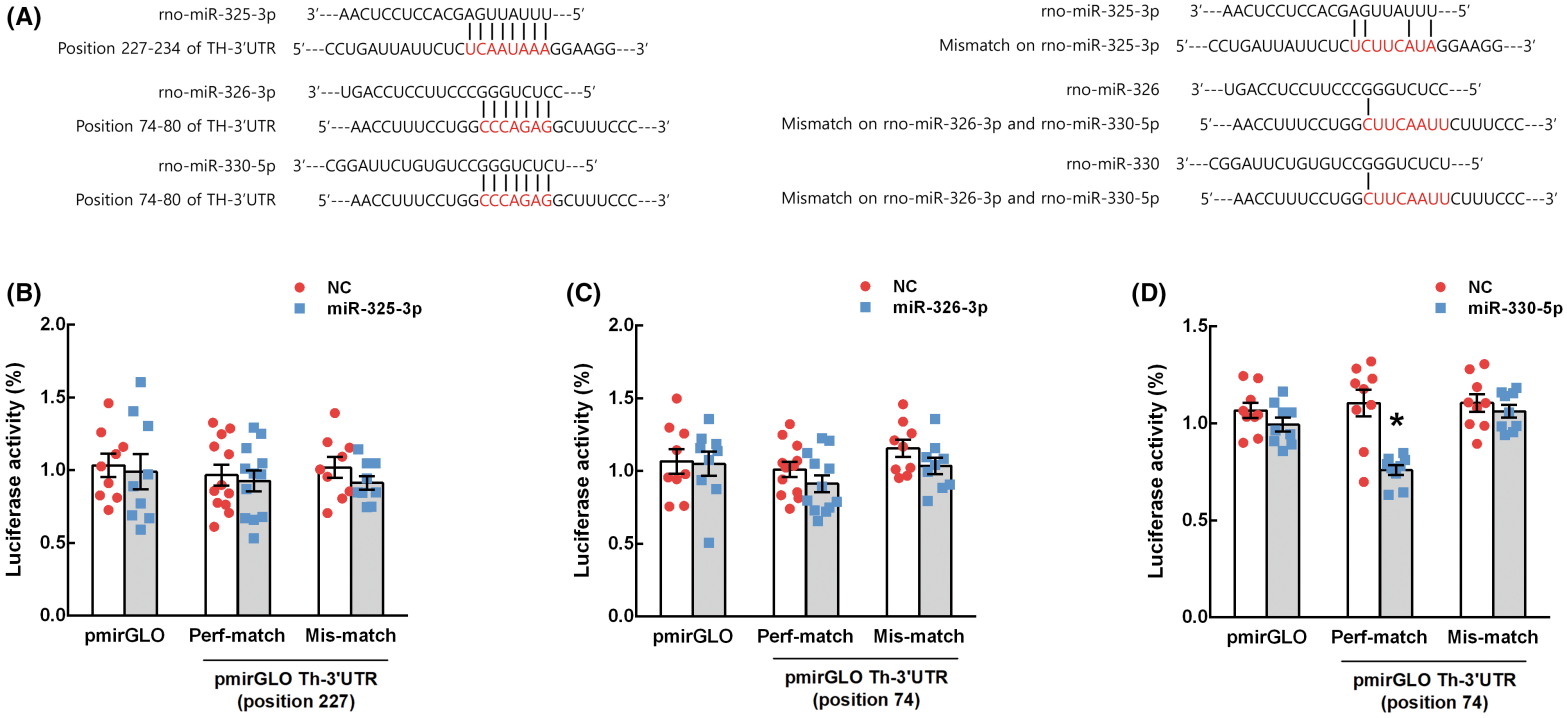
Binding activity of miR‐325‐3p, miR‐326‐3p, and miR‐330‐5p on the 3′‐untranslated region (UTR) of tyrosine hydroxylase (Th). Binding sites of miR‐325‐3p, miR‐326‐3p, and miR‐330‐5p in the 3′‐UTR of Th were predicted using the TargetScan v8.0 database (https://www.targetscan.org/vert_80/). (A) Perfect‐matched or mis‐matched segments were inserted into the 3′‐UTR of pmirGLO reporter vector. Mimics of miRNAs and the pmirGLO constructs were transfected into HEK‐293 cells. The direct interactions of miR‐325‐3p (B), miR‐326‐3p (C), and miR‐330‐5p (D) with predicted target sites were measured using a luciferase reporter assay. The value for firefly luciferase activity was normalized to the value for Renilla luciferase activity by the cotransfected pRL‐TK vector. Independent experiments were repeated in quadruples. Results are presented as mean ± SEM. Differences between the negative control (NC) and miRNA mimics were compared via unpaired *t*‐test (*p < 0.05).

We examined whether miR‐325‐3p, miR‐326‐3p, and miR‐330‐5p could directly target TH using a luciferase reporter assay. The binding sites of miR‐325‐3p, miR‐326‐3p, and miR‐330‐5p in the 3′‐UTR of the Th gene were predicted using the TargetScan database (Figure 4A). miRNA mimics and perfect‐matched or mismatched *Th* vectors were transfected into HEK‐293 cells. As shown in Figure 4B–D; miR‐325‐3p and miR‐326‐3p mimics did not change the luciferase activity in cells containing perfect‐matched and mismatched Th vectors (*t* = 0.400, *p* = 0.693 and *t* = 1.244, *p* = 0.226, respectively). In contrast, the miR‐330‐5p mimic significantly reduced the luciferase activity of the perfect‐matched Th vector (*t* = 4.715, *p* = 0.001) but not the mismatched Th vector (Figure 4D). These results indicate that miR‐330‐5p directly binds to its target sites in the 3′‐UTR of Th, whereas miR‐325‐3p and miR‐336‐3p do not.

### 3.5 Effect of miR‐330‐5p on TH expression and manic‐like behaviors in SD rats

We investigated whether miR‐330‐5p represses TH expression in the prefrontal cortex of SD rats and alleviates manic‐like behaviors. First, to confirm whether TH inhibition can alleviate manic‐like behaviors in SD rats, α‐methyl‐para‐tyrosine (AMPT) (Sigma–Aldrich), a competitive inhibitor of TH, was used. The rats were randomly divided into control (*n* = 8), SD (*n* = 10), and AMPT‐treated SD groups (*n* = 10). AMPT (250 mg/kg, to AMPT‐treated SD group) or saline (to control and SD groups) was administered intraperitoneally 2 h before the end of 72‐h sleep deprivation. After 2 h, behavioral tests were performed. Then, the rats were sacrificed, and the prefrontal cortex from each rat were dissected out. The prefrontal cortex of the left and right sides was separately stored at −80°C (16 or 20 samples per group were obtained). Total RNA (*n* = 8 on con, and *n* = 10 on SD and AMPT‐treated SD) and protein (*n* = 8 on con and AMPT‐treated SD, and *n* = 10 on SD) from tissues were isolated for qPCR and western blotting, regardless of left or right sides.

**FIGURE 5:**
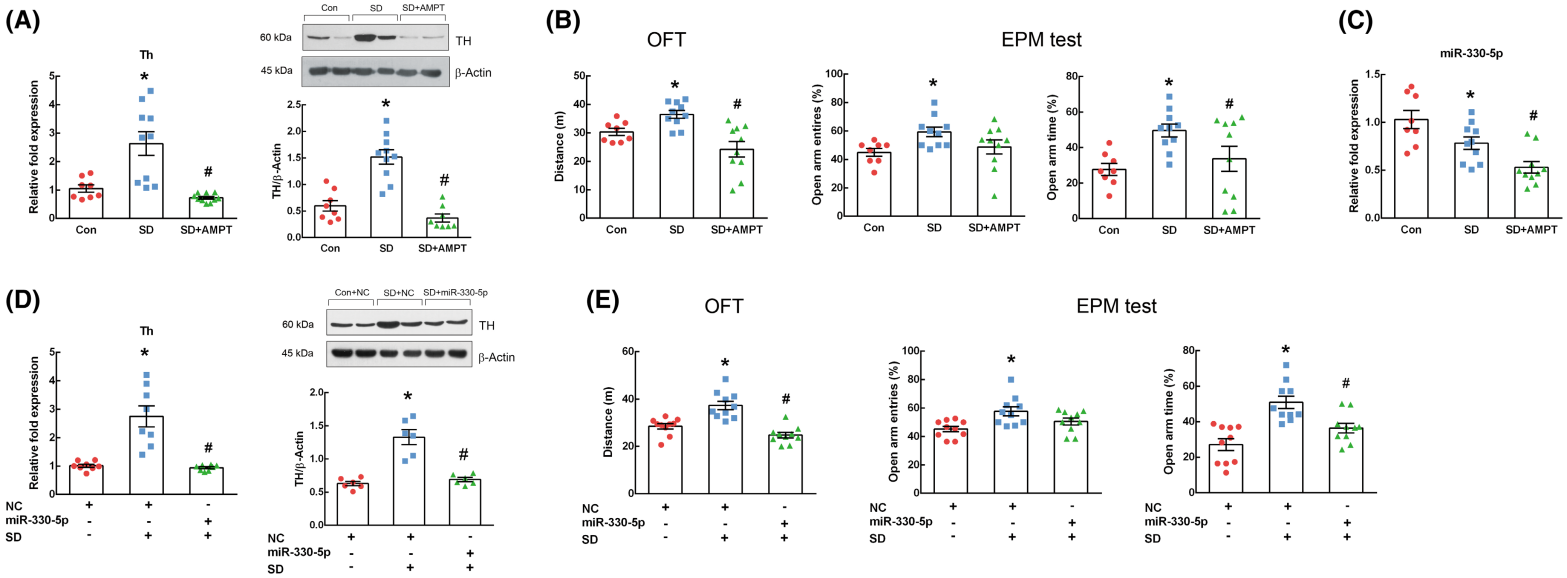
Effect of miR‐330‐5p on tyrosine hydroxylase (TH) expression and manic‐like behaviors in sleep‐deprived (SD) rats. TH mRNA and protein expressions were examined in the prefrontal cortex of SD rats treated with a TH inhibitor, α‐methyl‐para‐tyrosine (AMPT; 250 mg/kg, i.p.) (*n* = 8–10 per group) (A) and with miR‐330‐5p agomir (*n* = 8 per group) (D). Manic‐like behaviors were measured using the open field test and elevated plus maze (EPM) test in SD rats treated with AMPT (*n* = 8–10 per group) (B) and miR‐330‐5p agomir (*n* = 10 per group) (E). (C) In SD rats treated with AMPT, miR‐330‐5p expression was also assessed using quantitative PCR (qPCR) in the prefrontal cortex (*n* = 8–10 per group). Expression levels of mRNA and miRNA were normalized against those of Ubc and U87, respectively. β‐Actin expression was used as an internal control in western blotting. Results are shown as mean ± SEM. Differences between the groups were assessed by one‐way analysis of variance, followed by LSD post‐hoc test (*p < 0.05 compared to control treated with saline or negative control [NC] agomir, ^#^ *p* < 0.05 compared to SD treated with saline or NC agomir).

Figure 5A shows alteration in TH mRNA and protein expression in AMPT‐treated SD rats. A significant difference was detected in TH mRNA and protein expression (*F*_2,25_ = 15.173, *p* < 0.001 and *F*_2,23_ = 32.074, *p* < 0.001, respectively). Especially, AMPT inhibited TH mRNA and protein expression in the prefrontal cortex of SD rats (*p* < 0.001 on all in post‐hoc test). We also observed a difference in the travel distance between groups in the OFT (*F*_2,25_ = 10.169, *p* = 0.001; Figure 5B). In particular, an increase in the travel distance was detected in AMPT‐treated SD rats, compared to SD rats (*p* < 0.001). In the EPM test, significant differences between groups were found in both frequency of open‐arm entries (*F*_2,25_ = 3.487, *p* = 0.046) and time spent in the open arms (*F*_2,25_ = 4.688, *p* = 0.019). AMPT could alleviate the increase in time spent in the open arms in SD rats (*p* = 0.035 in post‐hoc test; Figure 5B), but not in the frequency of open‐arm entries (*p* > 0.05). Although AMPT did not reduce the frequency of open‐arm entries enhanced by SD, these results indicate that TH inhibition alleviates SD‐induced manic‐like behaviors such as hyperlocomotion and impulsivity. Additionally, we assessed the expression of miR‐330‐5p in the prefrontal cortex of AMPT‐treated SD rats. A significant difference in the expression of miR‐330‐5p was detected between groups (*F*_2,25_ = 11.574, *p* < 0.001). Expression of miR‐330‐5p was lower in AMPT‐treated SD rats than in SD rats (*p* = 0.016 in post‐hoc test; Figure 5C).

To examine the effect of miR‐330‐5p in SD rats, the rats were divided into three groups: control, SD, and miR‐330‐5p‐treated SD groups (*n* = 10 per group). The rats in the miR‐330‐5p‐treated SD group were treated with miR‐330‐5p agomir by ICV injection. NC agomir was injected into rats in the control and SD groups. After 72‐h SD and behavioral tests, the rats were sacrificed, and the prefrontal cortex was dissected out. The samples from the left and right sides of the brain were separately stored at −80°C. Finally, 20 samples per group were obtained. Eight samples per group for total RNA isolation and six samples per group for protein isolation were randomly used, regardless of left or right sides.

The expression levels of TH mRNA and protein were significantly different between groups (*F*_2,21_ = 22.210, *p* < 0.001 and *F*_2,15_ = 30.777, *p* < 0.001, respectively). As shown in Figure 5D, miR‐330‐5p agomir mitigated the increase in TH mRNA and protein expression induced by SD (*p* < 0.001 on both in post‐hoc test). In addition, we found a difference in the total travel distance between groups in OFT (*F*_2,27_ = 21.665, *p* < 0.001; Figure 5D). The miR‐330‐5p agomir significantly decreased the total travel distance in the OFT, compared to that in SD rats (*p* < 0.001). In the EPM test, the frequency of open‐arm entries, and the time spent in the open arms were significantly different between groups (*F*_2,27_ = 5.862, *p* = 0.008 and *F*_2,27_ = 14.254, *p* < 0.001, respectively). miR‐330‐5p agomir reduced the time spent in the open arms, compared to SD rats (*p* = 0.006), but not the number of open‐arm entries (Figure 5D). These results indicate that miR‐330‐5p alleviates manic‐like behavior in SD by suppressing TH expression.

## 4 DISCUSSION

Dysregulated dopamine transmission has been consistently implicated in BPD.^19^, ^20^, ^21^, ^22^, ^23^, ^34^ In particular, enhanced dopaminergic neurotransmission has been shown to facilitate mania. Indeed, during mania, the level of the dopamine metabolite homovanillic acid was elevated in the cerebrospinal fluid of patients in the manic phase of BPD.^20^, ^34^ In ouabain‐administered rats, which is a pharmacological mania model, TH activation has been shown in the striatum, together with hyperlocomotion.^23^ Additionally, in Slc6a3 knockdown mice, manic‐like behaviors were induced, and treatment with the TH inhibitor AMPT attenuated these behaviors.^22^

Enhanced dopaminergic neurotransmission has also been observed in SD rats. Increased levels of dopamine and its metabolite dihydroxyphenylacetic acid have been shown in various brain regions of rats subjected to SD for 72 or 96 h.^35^, ^36^, ^37^ A recent study measured dopamine levels in the brains of mice subjected to SD for 48–96 h and observed that dopamine levels increased in a time‐dependent manner, together with manic‐like behaviors.^36^ In our study, we found an increase in Th and Slc6a3 mRNA expression and a decrease in Drd1 and Drd2 mRNA expression in the prefrontal cortex of 72‐h SD rats, together with manic‐like behaviors such as hyperlocomotion and impulsivity. This increase in Th expression may indicate enhanced dopamine synthesis and release in the brains of SD rats. The increase in Slc6a3 expression and decrease in Drd1 and Drd2 expressions may be caused by enhanced dopamine synthesis and release. Previous studies have reported an increase in the binding activity and density of DRD1 and DRD2 in the brain of SD rats.^38^, ^39^, ^40^ The super‐sensitivity of dopamine receptors, together with the enhanced dopamine level, may lead to a decrease in Drd1 and Drd2 transcriptions in SD rats as a compensatory response. Focusing on TH, which showed the most potent alteration among the dopamine synapse genes, we predicted the target miRNAs of TH using the TargetScan database, based on DEmiRNAs identified in our pilot study, and finally selected miR‐325‐3p, miR‐336‐3p, and miR‐330‐5p (Table S9). We speculated that the attenuation of TH repression by miR‐325‐3p, miR‐336‐3p, and/or miR‐330‐5p might lead to increased TH levels in the prefrontal cortex of SD rats, inducing manic‐like behaviors. Therefore, we further examined this hypothesis.

To determine whether miR‐325‐3p, miR‐326‐3p, and miR‐330‐5p could suppress TH expression as target miRNAs, we assessed the effects of miRNAs mimics and inhibitors on TH mRNA and protein expression in Dex (5 and 50 μM)‐treated PC12 cells (with enhanced TH levels). At a low dose of Dex, miR‐325‐3p and miR‐326‐3p mimics alleviated the upregulation of Th mRNA expression, but not protein expression. However, at a high dose of Dex, neither TH mRNA nor protein expression was suppressed. Contrary to our expectation, miR‐325‐3p and miR‐326‐3p inhibitors suppressed Th mRNA expression in 5 μM Dex‐treated cells, similar to their mimics. Moreover, in Dex‐treated cells, miR‐325‐3p and miR‐326‐3p expression levels were upregulated, in addition to an increase in Th expression level (Figure 3A,B). In other words, the increase in TH expression concurred with the upregulation of miR‐325‐3p and miR‐326‐3p. These results indicate that miR‐325‐3p and miR‐336‐3p may not directly regulate TH expression as target miRNAs on TH. In contrast, the miR‐330‐5p mimic significantly repressed TH mRNA and protein expression at all doses of Dex. miR‐330‐5p inhibitor did not suppress TH expression. Moreover, we examined whether miRNAs could directly target TH by binding to the 3′‐UTR of Th using a luciferase reporter system. miR‐325‐3p and miR‐326‐3p mimics did not inhibit luciferase activity. In contrast, the miR‐330‐5p mimic attenuated luciferase activity compared to the NC mimic. These results indicate that miR‐330‐5p can directly repress TH expression by binding to its target sites in the 3′‐UTR of Th, whereas miR‐325‐3p and miR‐326‐3p cannot.

In SD rats, the suppression of TH may contribute to the alleviation of manic‐like behaviors. Based on the effects of miR‐330‐5p on TH, we examined whether miR‐330‐5p could suppress TH expression in the brains of SD rats, thus reducing manic‐like behaviors. First, we confirmed that TH inhibition decreased manic‐like behaviors in SD rats using AMPT. Previous clinical and experimental studies have reported that the administration of AMPT attenuates the severity of mania.^22^, ^41^, ^42^ Our results are also consistent with those of previous studies. Interestingly, ICV injection of the miR‐330‐5p agomir significantly decreased TH mRNA and protein expression in the prefrontal cortex of SD rats and reduced hyperlocomotion and impulsivity. These results indicate that miR‐330‐5p can reduce manic‐like behavior in SD rats by suppressing TH expression in the prefrontal cortex.

Intriguingly, in our results, miR‐330‐5p could regulate TH expression, but not be regulated by TH. Under an increase in TH expression following Dex treatment in PC12 cells, miR‐330‐5p expression did not change (Figure 3B). Under a decrease in TH expression by AMPT treatment in SD rats, miR‐330‐5p expression was reduced instead of being enhanced (Figure 5C). This suggests that sleep deprivation directly attenuates miR‐330‐5p expression, which in turn elevates TH expression. Consequently, although TH inhibition plays a major role in rescuing manic behaviors, its inhibition without modulating miRNA‐330‐5p may not completely relieve manic‐like behaviors in SD rats. In our study, we observed that the expression of miR‐330‐5p was lower in AMPT‐treated SD rats than in SD rats. Due to the persistent decrease in miR‐330‐5p, TH expression may be upregulated, at least partially, despite AMPT treatment; thus, the complete alleviation of manic‐like behaviors may be disturbed. In addition, we found that miR‐330‐5p did not suppress mRNA and protein expression of TH and caused behavioral change in control rats (Figure S2). miR‐330‐5p may not affect the basal level of TH and induce behavioral change in the normal state, not the manic state. Given these results, the regulation of TH through miR‐330‐5p may be a robust mechanism causing manic‐like behaviors in SD rats.

Dopamine plays an important role in movement, pleasure, motivation, and emotion. Altered activity of dopaminergic neurons is involved in the pathophysiology of various neurological and psychiatric diseases such as Parkinson's disease (PD), schizophrenia, and BPD. In addition, drugs that enhance or inhibit dopaminergic transmission have been used as major therapeutic strategies for these diseases. In schizophrenia, positive symptoms such as delusion, hallucination, and paranoia have been related to the hyperfunction of mesolimbic dopamine transmission.^43^, ^44^ Antipsychotic drugs, which antagonize DRD2 (with or without 5‐HT2A receptor inhibition), have been used against the positive symptoms of schizophrenia. Some antipsychotic drugs such as risperidone, chlorpromazine, and olanzapine are also used for the treatment of the manic phase of BPD. It may be involved in the inhibition of dopaminergic transmission enhanced in mania.^20^, ^22^, ^23^, ^34^ However, antipsychotic drugs can induce Parkinsonism as an adverse effect by excessive inhibition of dopamine transmission. PD is a progressive hypokinetic disease, and its motor symptoms are caused by dopamine deficit in the nigrostriatal pathway.^45^, ^46^ Thus, drugs enhancing dopaminergic transmission such as levodopa, and dopamine receptor agonists have been used for the alleviation of symptoms in PD. However, these drugs can cause psychosis as an adverse effect, enhancing dopamine transmission. In experimental studies, 1‐methyl‐4‐phenyl‐1,2,3,6‐tetrahydropyridine (MPTP), which is a neurotoxin used to produce experimental models of Parkinsonism, causes selective destruction of dopamine neurons in the substantia nigra. Especially, MPTP‐treated animals have shown a prominent reduction of TH in the striatum, along with impaired motor functions.^47^, ^48^, ^49^ Aznavour et al reported that MPTP‐induced parkinsonism was related to only decreased TH level, not SLC6A3.^47^ In contrast to PD animals, in manic‐like SD rats, we observed an increase of TH expression through the reduction of miR‐330‐5p in the prefrontal cortex. A previous study also showed an increase of TH expression in the nucleus accumbens of methamphetamine‐treated mice, which are considered a model for mania, schizophrenia, and addiction, showing enhanced synaptic dopamine levels.^50^ Interestingly, injection of dopaminergic toxin 6‐hydroxydopamine (6‐OHDA) antagonized the increase of TH in methamphetamine‐treated mice.^50^, ^51^, ^52^ 6‐OHDA is also a neurotoxin used to produce Parkinsonism rodent models.^53^, ^54^ Considering these previous reports and our results, the delicately balanced regulation of dopamine transmission including TH may play an important role in the drug therapy of PD, schizophrenia, and BPD. Given that miR‐330‐5p did not affect the basal level of TH (Figure 3C,E and Figure S2), the regulation of TH through miR‐330‐5p may be helpful in the alleviation of symptoms of these diseases, particularly in schizophrenia and BPD without concern about Parkinsonism. The involvement of the regulation of TH through miR‐330‐5p in PD, and schizophrenia may be needed to be further studied in animal models.

Many studies have explored alterations of miRNAs through the analysis of genome‐wide miRNA expression profiles,^13^, ^14^, ^15^, ^16^, ^17^, ^18^, ^55^, ^56^, ^57^, ^58^ and clinical implications of altered miRNAs in psychiatry diseases such as BPD and schizophrenia.^55^, ^57^ Beveridge et al showed significant upregulation of miR‐181b and downregulation of visinin‐like 1 (VSNL1) and glutamate ionotropic receptor AMPA type subunit 2 (GRIA2), which were target genes of miR‐181b, in schizophrenia postmortem brains.^55^ miR‐181b suppressed expressions of VSNL1 and GRIA2, via binding to miR‐181b‐recognition elements of those.^55^ Lai et al.^57^ identified 7 miRNAs (upregulated: miR‐34a, miR‐449a, miR‐564, miR‐58d, miR‐572, and miR‐652 and downregulated: miR‐432) associated with negative and cognitive symptoms of schizophrenia in PBMCs of patients with schizophrenia. They observed that the expressions of 7 miRNAs were not changed in PBMCs of patients with schizophrenia after 2 months of hospitalization, even with a significant improvement of clinical symptoms, suggesting the miRNAs as traits of schizophrenia, not just state‐dependent markers.^59^ Moreover, it has been suggested that several miRNAs are involved in the low incidence of malignancies in patients with schizophrenia.^60^ For instance, the expression level of miR‐183, which has been shown to activate the expression of tumor suppressor genes,^61^ was directly related to the absence of a solid tumor in patients with schizophrenia.^60^ In BPD, associations of several miRNAs such as miR‐185‐5p, miR‐142‐3p, and miR‐4286 have been observed in the peripheral blood or postmortem brains of patients with BPD.^14^, ^15^, ^16^, ^17^, ^18^ Through miRNA analysis of peripheral whole blood of patients with major depression (MD) or BPD, 5 miRNAs (let‐7a‐5p, let‐7d‐5p, let‐7f‐5p, miR‐24‐3p, and miR‐425‐3p) were specifically altered in patients with MD and 5 (miR‐140‐3p, miR‐30d‐5p, miR‐330‐5p, miR‐378a‐5p, and miR‐21‐3p) in patients with BD, whereas 2 miRNAs (miR‐330‐3p and miR‐345‐5p) were dysregulated in both diseases.^62^ Although the association of some miRNAs has been reported in psychiatric diseases, studies on the functions of the miRNAs on target genes and on the clinical implication of miRNAs are largely lacking. Future studies should focus on the roles of miRNAs on target genes. It may contribute to understanding the implications of miRNAs on the prognosis, and therapeutic response in psychiatric diseases.

In conclusion, our study showed that sleep deprivation increases manic‐like behaviors such as hyperlocomotion, impulsivity, and TH expression in the prefrontal cortex of rats. In addition, among the predicted target miRNAs on *Th*, miR‐325‐3p, miR‐326‐3p, and miR‐330‐5p, we found that miR‐330‐5p could directly repress TH by binding to its target sites in the 3′UTR of *Th*. Moreover, miR‐330‐5p alleviated the increase in TH expression and manic‐like behaviors in SD rats. Our results suggest that the regulation of TH via miR‐330‐5p may be implicated in the pathophysiology of mania in SD rats. We also found that expressions of *Gfap*, *Klh14*, *Fgd2*, and *Kcnip2*, which were miR‐330‐5p‐target genes predicted by the TargetScan v8.0 database, were reduced in miR‐330‐5p‐treated SD rats, compared to SD rats (Figure S3). Roles of these genes also needed to be elucidated in SD rats in further study, along with being regulated by miR‐330‐5p or not.

## AUTHOR CONTRIBUTIONS

HJP conceived and designed the study. All authors performed the experiments. KHL and HJP collected the data and performed data analysis. HJP drafted and finalized the manuscript. KHL edited the manuscript.

## CONFLICT OF INTEREST STATEMENT

All authors declare no conflict of interest.

## Supporting information


Appendix S1.

## Data Availability

The data that support the findings of this study are available from the corresponding author upon reasonable request.
