# Supplementary material for: Downregulation of microRNA‐330‐5p induces manic‐like behaviors in REM sleep‐deprived rats by enhancing tyrosine hydroxylase expression
Source: CNS Neurosci Ther. 2023 Feb 16;29(6):1525–36. doi: 10.1111/cns.14121 (PMC10173715; doi:10.1111/cns.14121)
Supplement: Supplementary file 1 — Appendix S1. [file CNS-29-1525-s001.zip › CNS_14121_Figure S1 (legend).docx]

**Figure S1.** Read length distribution of trimmed reads in miRNA-seq analysis. Con, control; SD, sleep deprivation.
