# Supplementary material for: Downregulation of microRNA‐330‐5p induces manic‐like behaviors in REM sleep‐deprived rats by enhancing tyrosine hydroxylase expression
Source: CNS Neurosci Ther. 2023 Feb 16;29(6):1525–36. doi: 10.1111/cns.14121 (PMC10173715; doi:10.1111/cns.14121)
Supplement: Supplementary file 1 — Appendix S1. [file CNS-29-1525-s001.zip › CNS_14121_revised supplementary materials_Leem et al_clean version.docx]

**RNA Isolation and RNA-Seq.** The next-generation sequencing was performed using the RNA pooled from 4 rats per group for screening candidate genes associated with SD. The total RNA was isolated from the prefrontal cortex samples pooled in each group using TRIzol. RNA integrity was measured using an Agilent 2100 Bioanalyzer (Agilent Technologies, Inc., Santa Clara, CA, USA). The cDNA libraries were constructed using the TruSeq RNA Library kit on 1 μg of total RNA according to the following process: extraction of polyA-selected RNA, fragmentation of RNA, reverse transcription using random hexamer, and 100 nt paired-end sequencing using Illumina HiSeq4000. We quantified the libraries through qPCR using an Agilent Technologies 2100 Bioanalyzer. The low quality and adapter sequences were excluded from the raw reads of the sequencer. And then, the reads were aligned to the Rattus norvegicus (UCSC rn6) using HISAT v2.0.5. The reference genome sequence of Rattus norvegicus and annotation data were acquired from the NCBI. The aligned reads were assembled into the transcripts, and their abundance was estimated using StringTie v1.3.3b. The relative abundance was estimated as fragments per kilobase of exon per million fragments mapped reads (FPKM) of transcript and gene expressed in each group. The RNAseq data has been uploaded to the public repository Sequence Read Archive (SRA) (https://submit.ncbi.nlm.nih.gov; SUB11956913).

**Statistical Analysis of Gene Expression Level.** To select differentially expressed genes (DEGs), the relative abundances for each gene were compared between groups. Genes whose FRKM was 0 in any of the groups were excluded from the analysis. After 1 was added to the FPKM values of genes, the values were converted based on log2 and then were subjected to quantile normalization. We determined the differential expression data by ∣fold change (FC)∣ ≥ 2 and independent t-test. To measure the similarity, hierarchical clustering for the DEG set was conducted using complete linkage and Euclidean distance. The gene-enrichment analysis for DEGs was also conducted based on the KEGG pathway (https://www.genome.jp/kegg/) database. Enrichment p values on KEGG pathway terms were calculated based on a modified Fisher’s exact test. And then, the false discovery rate (FDR-) adjusted *p* values using the Benjamini-Hochberg algorithm were calculated. All data analysis on RNA-seq was conducted using R 3.4.3 (<http://www.r-project.org/>).

**Altered Gene Expression in the Prefrontal Cortex of SD Rats.** To identify the alteration of gene expressions by SD, we performed RNA-seq in the prefrontal cortex of control and SD rats. Through RNA-seq, a total of 67,782,419 and 57,424,761 raw sequence reads from control and SD groups, respectively, were generated (Table S1). After quality filtering, we obtained 58,646,576 and 65,349,158 reads from control and SD groups. Of the filtered reads, 95.54 % (control) and 96.48% (SD) were mapped back to the reference Rattus norvegicus genome (UCSC rn6) (Table S2).

Finally, we acquired the expression data on 13,446 genes. In the DEG analysis on RNA-seq result, we selected genes showing ∣FC∣ ≥ 2.0 and the *p* value < 0.05 in comparison between control and SD. A total of 526 DEGs were identified in the control vs. SD comparison. The expressions of 216 genes were downregulated compared to control rats (Table S3), and the expressions of 310 genes were upregulated in SD rats (Table S4). A gene-enrichment analysis on 526 DEGs was performed, based on the KEGG pathway database. Fifty-five significant pathways were detected (FDR-corrected *p* < 0:05; Table S5). In addition, Table S6 shows genes belonging to neurotransmitter synapse pathways showing significant *p*-value in gene-enrichment analysis.

**Table S1.** mRNA-seq raw data generated and data analysis results.

| Sample name | Total read bases | Total reads | GC(%) | % of data > Q20 | % of data > Q30 |
| --- | --- | --- | --- | --- | --- |
| Con | 6,036,030,882 | 59,762,682 | 49.36 | 98.37 | 95.79 |
| SD | 6,718,452,734 | 66,519,334 | 49.48 | 98.45 | 95.96 |

**Table S2.** Read alignment summary in mRNA-seq

| Sample name | No. of processed reads | No. of mapped reads (%) |
| --- | --- | --- |
| Con | 58,646,576 | 56,616,386  (96.54%) |
| SD | 65,349,158 | 63,045,957  (96.48%) |

**Table S3.** Downregulated expressions of DEGs in the prefrontal cortex of sleep deprived (SD) rats

| Gene | Transcript ID | Description | FC |
| --- | --- | --- | --- |
|  |  |  | SD/Con |
| Myl6 | NM_001109484 | myosin light chain 6 | -27.373 |
| Myh9l1 | NM_013194 | myosin, heavy chain 9, non-muscle-like 1 | -10.404 |
| Trim54 | NM_001013217 | tripartite motif-containing 54 | -5.833 |
| Gpr88 | NM_031696 | G-protein coupled receptor 88 | -5.254 |
| Robo3 | NM_001108135 | roundabout guidance receptor 3 | -5.192 |
| Scn4b | NM_001008880 | sodium voltage-gated channel beta subunit 4 | -5.110 |
| Lyzl4 | NM_001246183 | lysozyme-like 4 | -5.081 |
| Nxph3 | NM_021679 | neurexophilin 3 | -4.914 |
| Pdyn | NM_019374 | prodynorphin | -4.890 |
| Tnnc2 | NM_001037351 | troponin C2, fast skeletal type | -4.220 |
| Sstr2 | NM_019348 | somatostatin receptor 2 | -4.137 |
| Trpv6 | NM_053686 | transient receptor potential cation channel, subfamily V, member 6 | -4.113 |
| Atoh7 | NM_001170482 | atonal bHLH transcription factor 7 | -4.045 |
| Krt2 | NM_001008899 | keratin 2 | -3.963 |
| Ucma | NM_001106121 | upper zone of growth plate and cartilage matrix associated | -3.771 |
| Rn45s | NR_046239 | 45S pre-ribosomal RNA | -3.752 |
| Satb2 | NM_001109306 | SATB homeobox 2 | -3.647 |
| Plk5 | NM_001170557 | polo-like kinase 5 | -3.630 |
| Aldh3b1 | NM_001006998 | aldehyde dehydrogenase 3 family, member B1 | -3.536 |
| Cox6a2 | NM_012812, NR_037674 | cytochrome c oxidase subunit 6A2 | -3.487 |
| Sprr1a | NM_021864 | small proline-rich protein 1A | -3.470 |
| Necab3 | NM_001098724 | N-terminal EF-hand calcium binding protein 3 | -3.440 |
| Ppp1r1b | NM_138521 | protein phosphatase 1, regulatory (inhibitor) subunit 1B | -3.424 |
| Sncg | NM_031688 | synuclein, gamma | -3.407 |
| P4ha3 | NM_198775 | prolyl 4-hydroxylase subunit alpha 3 | -3.402 |
| Olr59 | NM_173293 | olfactory receptor 59 | -3.325 |
| Fezf2 | NM_001107251 | Fez family zinc finger 2 | -3.321 |
| Pvalb | NM_022499 | parvalbumin | -3.234 |
| Rspo2 | NM_001130575 | R-spondin 2 | -3.228 |
| Ephb6 | NM_001107857 | Eph receptor B6 | -3.195 |
| Cyp11b1 | NM_012537 | cytochrome P450, family 11, subfamily b, polypeptide 1 | -3.175 |
| Neurod6 | NM_001109237 | neuronal differentiation 6 | -3.158 |
| Cacna2d3 | NM_175595 | calcium voltage-gated channel auxiliary subunit alpha2delta 3 | -3.157 |
| Arhgap25 | NM_001109247 | Rho GTPase activating protein 25 | -3.135 |
| Adgrl2 | NM_001190475, NM_001302208, NM_001302209, NM_001302210, NM_001302211, NM_001302212, NM_134408 | adhesion G protein-coupled receptor L2 | -3.117 |
| LOC100911717 | NR_132640 | uncharacterized LOC100911717 | -3.096 |
| Cyp26b1 | NM_181087 | cytochrome P450, family 26, subfamily b, polypeptide 1 | -3.096 |
| Prss22 | NM_001106984 | protease, serine, 22 | -3.088 |
| Dkk3 | NM_138519 | dickkopf WNT signaling pathway inhibitor 3 | -3.053 |
| Ramp3 | NM_020100 | receptor (G protein-coupled) activity modifying protein 3 | -3.050 |
| Hipk4 | NM_001024776 | homeodomain interacting protein kinase 4 | -3.009 |
| Kcns1 | NM_053954 | potassium voltage-gated channel, modifier subfamily S, member 1 | -2.961 |
| Abcg2 | NM_181381 | ATP-binding cassette, subfamily G (WHITE), member 2 | -2.924 |
| Myl4 | NM_001109495 | myosin, light chain 4 | -2.892 |
| Serinc2 | NM_001031656 | serine incorporator 2 | -2.886 |
| LOC257642 | NM_147136 | rRNA promoter binding protein | -2.851 |
| Neu2 | NM_017130 | neuraminidase 2 | -2.833 |
| LOC100125366 | NR_144446 | 40S ribosomal protein S2 | -2.814 |
| Kcnt2 | NM_198762 | potassium sodium-activated channel subfamily T member 2 | -2.810 |
| Cd34 | NM_001107202 | CD34 molecule | -2.781 |
| Gpr83 | NM_080411 | G protein-coupled receptor 83 | -2.779 |
| Tshz3 | NM_001107506 | teashirt zinc finger homeobox 3 | -2.755 |
| Dact2 | NM_001107464 | dapper, antagonist of beta-catenin, homolog 2 (Xenopus laevis) | -2.747 |
| LOC691153 | NM_001109627 | hypothetical protein LOC691153 | -2.731 |
| Drd1 | NM_012546 | dopamine receptor D1 | -2.724 |
| Slc30a3 | NM_001013243 | solute carrier family 30 member 3 | -2.719 |
| Lamp5 | NM_001014183 | lysosomal-associated membrane protein family, member 5 | -2.711 |
| Bhmt | NM_030850 | betaine-homocysteine S-methyltransferase | -2.701 |
| Pcsk2 | NM_012746 | proprotein convertase subtilisin/kexin type 2 | -2.696 |
| Cplx3 | NM_001109295 | complexin 3 | -2.691 |
| Pcdha12 | NM_053940 | protocadherin alpha 12 | -2.688 |
| Sstr1 | NM_012719 | somatostatin receptor 1 | -2.686 |
| Htr6 | NM_024365 | 5-hydroxytryptamine receptor 6 | -2.673 |
| Igfbp6 | NM_013104 | insulin-like growth factor binding protein 6 | -2.662 |
| Pou3f1 | NM_138838 | POU class 3 homeobox 1 | -2.661 |
| Oprk1 | NM_001318742, NM_017167 | opioid receptor, kappa 1 | -2.657 |
| Htatip2 | NM_001106263 | HIV-1 Tat interactive protein 2 | -2.653 |
| Gfra1 | NM_012959 | GDNF family receptor alpha 1 | -2.646 |
| Cobl | NM_001107236 | cordon-bleu WH2 repeat protein | -2.637 |
| Cyp11b2 | NM_012538 | cytochrome P450, family 11, subfamily b, polypeptide 2 | -2.630 |
| Vamp1 | NM_013090 | vesicle-associated membrane protein 1 | -2.628 |
| Dnajb13 | NM_001005885 | DnaJ heat shock protein family (Hsp40) member B13 | -2.628 |
| Ovol2 | NM_001106519 | ovo-like zinc finger 2 | -2.626 |
| Hapln4 | NM_001108398 | hyaluronan and proteoglycan link protein 4 | -2.600 |
| Perp | NM_001106265 | PERP, TP53 apoptosis effector | -2.585 |
| Tspan17 | NM_001013138 | tetraspanin 17 | -2.573 |
| Pcsk4 | NM_133559 | proprotein convertase subtilisin/kexin type 4 | -2.564 |
| Adra1b | NM_016991 | adrenoceptor alpha 1B | -2.557 |
| RGD1561149 | NM_001134629 | similar to mKIAA1522 protein | -2.555 |
| Nrgn | NM_024140 | neurogranin | -2.551 |
| Moap1 | NM_001013101 | modulator of apoptosis 1 | -2.529 |
| Ldb2 | NM_001106009 | LIM domain binding 2 | -2.521 |
| Trpm4 | NM_001136229 | transient receptor potential cation channel, subfamily M, member 4 | -2.520 |
| Lhx6 | NM_001107837 | LIM homeobox 6 | -2.514 |
| Hpca | NM_017122 | hippocalcin | -2.510 |
| Kcns3 | NM_031778 | potassium voltage-gated channel, modifier subfamily S, member 3 | -2.494 |
| Rassf7 | NM_001106317 | Ras association domain family member 7 | -2.491 |
| Chrna5 | NM_017078 | cholinergic receptor nicotinic alpha 5 subunit | -2.459 |
| Tmem178a | NM_001004282 | transmembrane protein 178A | -2.451 |
| Pla2g2d | NM_001013428 | phospholipase A2, group IID | -2.437 |
| Pgr | NM_022847 | progesterone receptor | -2.436 |
| Drd2 | NM_012547 | dopamine receptor D2 | -2.431 |
| Sertm1 | NM_001109580 | serine-rich and transmembrane domain containing 1 | -2.427 |
| B3galt2 | NM_001109492 | Beta-1,3-galactosyltransferase 2 | -2.424 |
| Bmper | NM_001135799 | BMP-binding endothelial regulator | -2.419 |
| Lingo2 | NM_001107926 | leucine rich repeat and Ig domain containing 2 | -2.419 |
| Gypc | NM_001013233 | glycophorin C (Gerbich blood group) | -2.413 |
| Nptx1 | NM_153735 | neuronal pentraxin 1 | -2.412 |
| LOC100910996 | NM_001271382 | uncharacterized LOC100910996 | -2.374 |
| Prph | NM_012633 | peripherin | -2.365 |
| Lrrc10b | NM_001107577 | leucine rich repeat containing 10B | -2.361 |
| Kcnk4 | NM_053804 | potassium two pore domain channel subfamily K member 4 | -2.357 |
| Rtn4r | NM_053613 | reticulon 4 receptor | -2.356 |
| Syt2 | NM_012665 | synaptotagmin 2 | -2.349 |
| Rassf5 | NM_019365 | Ras association domain family member 5 | -2.346 |
| Galnt14 | NM_001012109 | polypeptide N-acetylgalactosaminyltransferase 14 | -2.333 |
| Lypd6b | NM_001134580 | LY6/PLAUR domain containing 6B | -2.333 |
| Tmem114 | NM_001134638 | transmembrane protein 114 | -2.326 |
| Flt3 | NM_001100822 | fms-related tyrosine kinase 3 | -2.321 |
| Vipr1 | NM_012685 | vasoactive intestinal peptide receptor 1 | -2.313 |
| Nrn1 | NM_053346 | neuritin 1 | -2.310 |
| Susd2 | NM_001106381 | sushi domain containing 2 | -2.304 |
| Meltf | NM_001105872 | melanotransferrin | -2.299 |
| Lrrc57 | NM_001012354 | leucine rich repeat containing 57 | -2.288 |
| Vsig2 | NM_001106812 | V-set and immunoglobulin domain containing 2 | -2.281 |
| Cplx2 | NM_053878 | complexin 2 | -2.280 |
| Kcng4 | NM_001107435 | potassium voltage-gated channel modifier subfamily G member 4 | -2.276 |
| Thpo | NM_031133 | thrombopoietin | -2.274 |
| Car8 | NM_001009662 | carbonic anhydrase 8 | -2.265 |
| Lmntd2 | NM_001127540 | lamin tail domain containing 2 | -2.240 |
| Rims1 | NM_052829 | regulating synaptic membrane exocytosis 1 | -2.233 |
| Trpc6 | NM_053559 | transient receptor potential cation channel, subfamily C, member 6 | -2.230 |
| Acot4 | NM_001109440 | acyl-CoA thioesterase 4 | -2.228 |
| Vsnl1 | NM_012686 | visinin-like 1 | -2.223 |
| Zdhhc22 | NM_001039325 | zinc finger, DHHC-type containing 22 | -2.220 |
| Wnt10a | NM_001108227 | wingless-type MMTV integration site family, member 10A | -2.220 |
| Tmem14a | NM_001108790 | transmembrane protein 14A | -2.217 |
| Lpin3 | NM_001014184 | lipin 3 | -2.216 |
| Sst | NM_012659 | somatostatin | -2.210 |
| Fxyd7 | NM_022008 | FXYD domain-containing ion transport regulator 7 | -2.208 |
| Hp | NM_012582 | haptoglobin | -2.204 |
| Kcnc2 | NM_139216, NM_139217 | potassium voltage-gated channel subfamily C member 2 | -2.203 |
| LOC100125362 | NM_001103354 | hypothetical protein LOC100125362 | -2.200 |
| Slc24a2 | NM_031743 | solute carrier family 24 member 2 | -2.198 |
| Asah2 | NM_053646 | N-acylsphingosine amidohydrolase (non-lysosomal ceramidase) 2 | -2.194 |
| Ccl17 | NM_057151 | C-C motif chemokine ligand 17 | -2.193 |
| Scn1b | NM_001271045, NM_001271046, NM_017288 | sodium voltage-gated channel beta subunit 1 | -2.193 |
| Rbm11 | NM_001105898 | RNA binding motif protein 11 | -2.191 |
| Vip | NM_053991 | vasoactive intestinal peptide | -2.191 |
| Slc41a2 | NM_001108742 | solute carrier family 41 member 2 | -2.188 |
| Npas1 | NM_001107479 | neuronal PAS domain protein 1 | -2.187 |
| Inpp5d | NM_019311 | inositol polyphosphate-5-phosphatase D | -2.184 |
| Pstpip1 | NM_001106824 | proline-serine-threonine phosphatase-interacting protein 1 | -2.178 |
| Gpr68 | NM_001108049 | G protein-coupled receptor 68 | -2.176 |
| Slc22a3 | NM_019230 | solute carrier family 22 member 3 | -2.171 |
| Dclk3 | NM_001191800 | doublecortin-like kinase 3 | -2.170 |
| Hs3st2 | NM_181370 | heparan sulfate-glucosamine 3-sulfotransferase 2 | -2.169 |
| Kcnk2 | NM_172041, NM_172042 | potassium two pore domain channel subfamily K member 2 | -2.169 |
| Ephx4 | NM_001105994 | epoxide hydrolase 4 | -2.168 |
| Thrsp | NM_012703 | thyroid hormone responsive | -2.166 |
| F12 | NM_001014006 | coagulation factor XII | -2.165 |
| Fap | NM_138850 | fibroblast activation protein, alpha | -2.155 |
| Hcrtr1 | NM_013064 | hypocretin receptor 1 | -2.155 |
| Ccdc42 | NM_001107009 | coiled-coil domain containing 42 | -2.151 |
| Treml1 | NM_001192001 | triggering receptor expressed on myeloid cells-like 1 | -2.149 |
| Ttc22 | NM_001106671 | tetratricopeptide repeat domain 22 | -2.144 |
| Fhl2 | NM_031677 | four and a half LIM domains 2 | -2.136 |
| Cnksr2 | NM_001113366, NM_021686 | connector enhancer of kinase suppressor of Ras 2 | -2.131 |
| Olfm3 | NM_145777 | olfactomedin 3 | -2.131 |
| Hrasls | NM_001105871 | HRAS-like suppressor | -2.126 |
| Lgals7 | NM_022582 | galectin 7 | -2.122 |
| Grp | NM_133570 | gastrin releasing peptide | -2.115 |
| Phyhip | NM_001017376 | phytanoyl-CoA 2-hydroxylase interacting protein | -2.114 |
| Rasl10a | NM_001108862 | RAS-like, family 10, member A | -2.111 |
| Eps8l2 | NM_001108508 | EPS8-like 2 | -2.110 |
| Lrrk2 | NM_001191789 | leucine-rich repeat kinase 2 | -2.109 |
| Pcdha3 | NM_053941 | protocadherin alpha 3 | -2.109 |
| Stx1a | NM_053788 | syntaxin 1A | -2.107 |
| Nags | NM_001107053 | N-acetylglutamate synthase | -2.103 |
| Ebi3 | NM_001109421 | Epstein-Barr virus induced 3 | -2.093 |
| Rims3 | NM_022931 | regulating synaptic membrane exocytosis 3 | -2.086 |
| Ackr2 | NM_078621 | atypical chemokine receptor 2 | -2.085 |
| Dmgdh | NM_139102 | dimethylglycine dehydrogenase | -2.083 |
| Rnf39 | NM_134374 | ring finger protein 39 | -2.082 |
| Slc4a11 | NM_001107775 | solute carrier family 4 member 11 | -2.077 |
| Entpd3 | NM_178106 | ectonucleoside triphosphate diphosphohydrolase 3 | -2.071 |
| Aptr | NR_130137 | Alu-mediated CDKN1A/p21 transcriptional regulator | -2.069 |
| Rph3a | NM_133518 | rabphilin 3A | -2.066 |
| Tbr1 | NM_001191070 | T-box, brain, 1 | -2.064 |
| Chrm3 | NM_012527 | cholinergic receptor, muscarinic 3 | -2.061 |
| Prrt2 | NM_001276470, NR_077057 | proline-rich transmembrane protein 2 | -2.061 |
| LOC100911827 | NR_110710 | uncharacterized LOC100911827 | -2.060 |
| Itpr1 | NM_001007235, NM_001270596, NM_001270597 | inositol 1,4,5-trisphosphate receptor, type 1 | -2.059 |
| Abcd2 | NM_033352 | ATP binding cassette subfamily D member 2 | -2.057 |
| Mmel1 | NM_001107997 | membrane metallo-endopeptidase-like 1 | -2.056 |
| Hsd17b13 | NM_001009684 | hydroxysteroid (17-beta) dehydrogenase 13 | -2.056 |
| Prr15 | NM_001104527 | proline rich 15 | -2.056 |
| Ltk | NM_001107763 | leukocyte receptor tyrosine kinase | -2.054 |
| Cbln2 | NM_001012740 | cerebellin 2 precursor | -2.053 |
| Atp13a5 | NM_001191657 | ATPase 13A5 | -2.052 |
| Gpr6 | NM_031806 | G protein-coupled receptor 6 | -2.051 |
| Nptxr | NM_030841 | neuronal pentraxin receptor | -2.049 |
| Orai2 | NM_001170403 | ORAI calcium release-activated calcium modulator 2 | -2.047 |
| Pnoc | NM_013007 | prepronociceptin | -2.045 |
| Pamr1 | NM_001107755 | peptidase domain containing associated with muscle regeneration 1 | -2.036 |
| Dok5 | NM_001109344 | docking protein 5 | -2.028 |
| Rasd2 | NM_133568 | RASD family, member 2 | -2.027 |
| Sptssb | NM_001271299, NM_001271301 | serine palmitoyltransferase, small subunit B | -2.026 |
| Rundc3b | NM_001047116 | RUN domain containing 3B | -2.025 |
| Wnt4 | NM_053402 | wingless-type MMTV integration site family, member 4 | -2.022 |
| Slc2a13 | NM_133611 | solute carrier family 2 member 13 | -2.022 |
| Upk2 | NM_001109523 | uroplakin 2 | -2.021 |
| Mir1249 | NR_037390 | microRNA 1249 | -2.019 |
| Fam107a | NM_001025129 | family with sequence similarity 107, member A | -2.016 |
| Bc1 | NR_036653 | brain cytoplasmic RNA 1 | -2.014 |
| Calhm2 | NM_001008306 | calcium homeostasis modulator 2 | -2.014 |
| Stac2 | NM_001108834 | SH3 and cysteine rich domain 2 | -2.012 |
| Itpka | NM_031045 | inositol-trisphosphate 3-kinase A | -2.011 |
| Ntng2 | NM_001107825 | netrin G2 | -2.011 |
| Sv2b | NM_057207 | synaptic vesicle glycoprotein 2b | -2.011 |
| Trpc5 | NM_080898 | transient receptor potential cation channel, subfamily C, member 5 | -2.010 |
| Ngef | NM_001136241 | neuronal guanine nucleotide exchange factor | -2.009 |
| Myo1b | NM_053986 | myosin Ib | -2.008 |
| E2f1 | NM_001100778 | E2F transcription factor 1 | -2.006 |
| Olfm2 | NM_001015017 | olfactomedin 2 | -2.006 |
| Agbl4 | NM_001350232 | ATP/GTP binding protein-like 4 | -2.004 |

FC, fold change

**Table S4.** Upregulated expressions of DEGs in the prefrontal cortex of sleep deprived (SD) rats

| Gene | Transcript ID | Description | FC |
| --- | --- | --- | --- |
|  |  |  | SD/Con |
| Cela3b | NM_001106692 | chymotrypsin-like elastase family, member 3B | 2.000 |
| C1r | NM_001134555 | complement C1r subcomponent | 2.003 |
| Fgd3 | NM_001108409 | FYVE, RhoGEF and PH domain containing 3 | 2.005 |
| Sncaip | NM_001107379 | synuclein, alpha interacting protein | 2.006 |
| Ncbp3 | NM_001108281 | nuclear cap binding subunit 3 | 2.007 |
| Abca8a | NM_001281824 | ATP-binding cassette, subfamily A (ABC1), member 8a | 2.007 |
| Homer2 | NM_053309 | homer scaffolding protein 2 | 2.009 |
| Trhr | NM_013047 | thyrotropin releasing hormone receptor | 2.011 |
| Elovl2 | NM_001109118 | ELOVL fatty acid elongase 2 | 2.016 |
| Ube2l6 | NM_001024755 | ubiquitin-conjugating enzyme E2L 6 | 2.017 |
| Sec31b | NM_001135713 | SEC31 homolog B, COPII coat complex component | 2.024 |
| Mir337 | NR_031781 | microRNA 337 | 2.027 |
| Cdh1 | NM_031334 | cadherin 1 | 2.036 |
| Nav2 | NM_138529 | neuron navigator 2 | 2.041 |
| Alox15b | NM_153301 | arachidonate 15-lipoxygenase, type B | 2.042 |
| Ptch2 | NM_001108975 | patched 2 | 2.042 |
| H3f3b | NM_053985 | H3 histone, family 3B | 2.044 |
| Kcnh2 | NM_053949 | potassium voltage-gated channel subfamily H member 2 | 2.045 |
| Htr5b | NM_024395 | 5-hydroxytryptamine (serotonin) receptor 5B | 2.046 |
| Rgs16 | NM_001077589 | regulator of G-protein signaling 16 | 2.048 |
| Kcnip2 | NM_001033961, NM_020094, NM_020095 | Kv channel-interacting protein 2 | 2.054 |
| Cacna1i | NM_020084 | calcium voltage-gated channel subunit alpha1 I | 2.056 |
| Noct | NM_138526 | nocturnin | 2.066 |
| Ier2 | NM_001009541 | immediate early response 2 | 2.069 |
| P3h3 | NM_001106620 | prolyl 3-hydroxylase 3 | 2.070 |
| Kcne5 | NM_001101003 | potassium voltage-gated channel subfamily E regulatory subunit 5 | 2.073 |
| Myt1 | NM_001108615 | myelin transcription factor 1 | 2.077 |
| Alox15 | NM_031010 | arachidonate 15-lipoxygenase | 2.078 |
| Fam207a | NM_001008307 | family with sequence similarity 207, member A | 2.084 |
| Fam126a | NM_001191969 | family with sequence similarity 126, member A | 2.084 |
| LOC691995 | NM_001103353 | hypothetical protein LOC691995 | 2.087 |
| Apoc4 | NM_001109419 | apolipoprotein C4 | 2.103 |
| Efnb3 | NM_001100980 | ephrin B3 | 2.104 |
| Cebpd | NM_013154 | CCAAT/enhancer binding protein delta | 2.105 |
| Kcnip1 | NM_001261387, NM_001261388, NM_001261389, NM_022929 | potassium voltage-gated channel interacting protein 1 | 2.106 |
| Htr1f | NM_021857 | 5-hydroxytryptamine receptor 1F | 2.107 |
| Cited2 | NM_053698 | Cbp/p300-interacting transactivator, with Glu/Asp-rich carboxy-terminal domain, 2 | 2.110 |
| Sez6 | NM_001105754 | seizure related 6 homolog | 2.114 |
| Cd74 | NM_013069 | CD74 molecule | 2.116 |
| Vwce | NM_001271311 | von Willebrand factor C and EGF domains | 2.122 |
| Mir568 | NR_032745 | microRNA 568 | 2.123 |
| Slc6a3 | NM_012694 | solute carrier family 6 member 3 | 2.129 |
| Hist1h4b | NM_022686 | histone cluster 1, H4b | 2.132 |
| Adarb2 | NM_133302 | adenosine deaminase, RNA-specific, B2 | 2.138 |
| Vim | NM_031140 | vimentin | 2.139 |
| Mia | NM_030852 | melanoma inhibitory activity | 2.146 |
| Midn | NM_001191577 | midnolin | 2.149 |
| Ybx1 | NM_031563 | Y box binding protein 1 | 2.155 |
| Fibin | NM_001025042 | fin bud initiation factor homolog (zebrafish) | 2.155 |
| Fam26e | NM_001024977 | family with sequence similarity 26, member E | 2.159 |
| Adcyap1r1 | NM_001270579, NM_001270580, NM_001270581, NM_001270582, NM_001270583, NM_133511 | adenylate cyclase activating polypeptide 1 receptor type 1 | 2.165 |
| Synpo2 | NM_001191963 | synaptopodin 2 | 2.165 |
| Tia1 | NM_001012096 | TIA1 cytotoxic granule-associated RNA binding protein | 2.166 |
| Zeb1 | NM_001308265 | zinc finger E-box binding homeobox 1 | 2.168 |
| Igsf3 | NM_001106455 | immunoglobulin superfamily, member 3 | 2.169 |
| Atp1a4 | NM_001271030, NM_022848 | ATPase Na+/K+ transporting subunit alpha 4 | 2.172 |
| Hba1 | NM_013096 | hemoglobin, alpha 1 | 2.174 |
| Fam20c | NM_001012238 | family with sequence similarity 20, member C | 2.187 |
| Nradd | NM_139259 | neurotrophin receptor associated death domain | 2.188 |
| Ret | NM_001110099, NM_012643 | ret proto-oncogene | 2.189 |
| Pipox | NM_001012009 | pipecolic acid and sarcosine oxidase | 2.190 |
| Tmod2 | NM_031613 | tropomodulin 2 | 2.194 |
| Col5a2 | NM_053488 | collagen type V alpha 2 chain | 2.194 |
| Ankrd29 | NM_001190372 | ankyrin repeat domain 29 | 2.199 |
| Ppp1r1c | NM_001109200 | protein phosphatase 1, regulatory (inhibitor) subunit 1C | 2.200 |
| C1s | NM_138900 | complement component 1, s subcomponent | 2.200 |
| Ptgds | NM_013015 | prostaglandin D2 synthase | 2.202 |
| Mir212 | NR_031925 | microRNA 212 | 2.207 |
| Igf2 | NM_001190162, NM_001190163, NM_031511 | insulin-like growth factor 2 | 2.210 |
| Serping1 | NM_199093 | serpin family G member 1 | 2.212 |
| Csk | NM_001030039 | c-src tyrosine kinase | 2.217 |
| Ntsr1 | NM_001108967 | neurotensin receptor 1 | 2.221 |
| Hap1 | NM_024133, NM_177982 | huntingtin-associated protein 1 | 2.225 |
| Tfap2c | NM_201420 | transcription factor AP-2 gamma | 2.226 |
| Cox6b2 | NM_001039085 | cytochrome c oxidase subunit VIb polypeptide 2 | 2.231 |
| Arhgef6 | NM_001005565 | Rac/Cdc42 guanine nucleotide exchange factor 6 | 2.233 |
| RT1-CE7 | NM_001008845 | RT1 class I, locus CE7 | 2.238 |
| Itgb4 | NM_013180 | integrin subunit beta 4 | 2.238 |
| Gfral | NM_001191998 | GDNF family receptor alpha like | 2.246 |
| Bmp7 | NM_001191856 | bone morphogenetic protein 7 | 2.247 |
| Nt5dc2 | NM_001009271 | 5'-nucleotidase domain containing 2 | 2.251 |
| Zufsp | NM_001008308 | zinc finger with UFM1-specific peptidase domain | 2.251 |
| Slc22a6 | NM_017224 | solute carrier family 22 member 6 | 2.252 |
| LOC689064 | NM_001111269 | beta-globin | 2.253 |
| St6galnac2 | NM_001031652 | ST6 N-acetylgalactosaminide alpha-2,6-sialyltransferase 2 | 2.258 |
| Grm4 | NM_022666 | glutamate metabotropic receptor 4 | 2.267 |
| Gfap | NM_017009 | glial fibrillary acidic protein | 2.268 |
| Ddx17 | NM_001015018 | DEAD-box helicase 17 | 2.268 |
| Thbs1 | NM_001013062 | thrombospondin 1 | 2.271 |
| Tgfa | NM_012671 | transforming growth factor alpha | 2.272 |
| Cnn3 | NM_019359 | calponin 3 | 2.286 |
| Sertad4 | NM_001108351 | SERTA domain containing 4 | 2.291 |
| Slc4a4 | NM_053424 | solute carrier family 4 member 4 | 2.293 |
| Col11a1 | NM_013117 | collagen type XI alpha 1 chain | 2.295 |
| Peli2 | NM_001107259 | pellino E3 ubiquitin protein ligase family member 2 | 2.298 |
| Casp4 | NM_053736 | caspase 4 | 2.300 |
| Sik1 | NM_021693 | salt-inducible kinase 1 | 2.302 |
| Wnt6 | NM_001108226 | wingless-type MMTV integration site family, member 6 | 2.306 |
| Scn7a | NM_031686 | sodium voltage-gated channel alpha subunit 7 | 2.318 |
| Kcnh6 | NM_053937 | potassium voltage-gated channel subfamily H member 6 | 2.332 |
| Amz1 | NM_001047092 | archaelysin family metallopeptidase 1 | 2.333 |
| Ctbp2 | NM_053335 | C-terminal binding protein 2 | 2.337 |
| RGD1565283 | NM_001109074 | similar to novel protein | 2.339 |
| Kcnk13 | NM_022293 | potassium two pore domain channel subfamily K member 13 | 2.339 |
| Trafd1 | NM_053760 | TRAF type zinc finger domain containing 1 | 2.339 |
| Rbp1 | NM_012733 | retinol binding protein 1 | 2.340 |
| Dclk2 | NM_001009691, NM_001195832 | doublecortin-like kinase 2 | 2.345 |
| Cd8a | NM_031538 | CD8a molecule | 2.349 |
| Itgam | NM_012711 | integrin subunit alpha M | 2.357 |
| Ntn4 | NM_001106780 | netrin 4 | 2.358 |
| Arc | NM_019361 | activity-regulated cytoskeleton-associated protein | 2.359 |
| Egr1 | NM_012551 | early growth response 1 | 2.360 |
| Usp18 | NM_001014058 | ubiquitin specific peptidase 18 | 2.363 |
| Isg15 | NM_001106700 | ISG15 ubiquitin-like modifier | 2.372 |
| Zfp608 | NM_001107378 | zinc finger protein 608 | 2.387 |
| Spry1 | NM_001106427 | sprouty RTK signaling antagonist 1 | 2.391 |
| Gstm2 | NM_177426 | glutathione S-transferase mu 2 | 2.394 |
| Zic1 | NM_022677 | Zic family member 1 | 2.395 |
| Dchs1 | NM_001107544 | dachsous cadherin-related 1 | 2.396 |
| Plppr3 | NM_181634 | phospholipid phosphatase related 3 | 2.400 |
| RT1-Ba | NM_001008831 | RT1 class II, locus Ba | 2.402 |
| Akna | NM_001108668 | AT-hook transcription factor | 2.414 |
| Slc32a1 | NM_031782 | solute carrier family 32 member 1 | 2.418 |
| Fabp4 | NM_053365 | fatty acid binding protein 4 | 2.436 |
| Inmt | NM_001109022 | indolethylamine N-methyltransferase | 2.437 |
| Cpne7 | NM_001108454 | copine 7 | 2.439 |
| LOC498675 | NM_001109113 | hypothetical LOC498675 | 2.443 |
| Baiap3 | NM_001312663 | BAI1-associated protein 3 | 2.446 |
| Slc47a1 | NM_001014118 | solute carrier family 47 member 1 | 2.451 |
| Ppm1e | NM_198773 | protein phosphatase, Mg2+/Mn2+ dependent, 1E | 2.463 |
| Fosl2 | NM_001013146, NM_012954 | FOS like 2, AP-1 transcription factor subunit | 2.467 |
| Sv2c | NM_031593 | synaptic vesicle glycoprotein 2c | 2.484 |
| Nr4a3 | NM_031628 | nuclear receptor subfamily 4, group A, member 3 | 2.484 |
| Tpsg1 | NM_175593 | tryptase gamma 1 | 2.484 |
| Cxxc1 | NM_001079698 | CXXC finger protein 1 | 2.489 |
| Efemp1 | NM_001012039 | EGF-containing fibulin-like extracellular matrix protein 1 | 2.495 |
| Khps1a | NR_133650 | sphk1a antisense transcript | 2.498 |
| Nos1 | NM_052799 | nitric oxide synthase 1 | 2.504 |
| Grid2ip | NM_001105910 | Grid2 interacting protein | 2.513 |
| Rassf8 | NM_001191753 | Ras association domain family member 8 | 2.518 |
| Serpinf1 | NM_177927 | serpin family F member 1 | 2.518 |
| Frzb | NM_001100527 | frizzled-related protein | 2.519 |
| Grin3b | NM_133308 | glutamate ionotropic receptor NMDA type subunit 3B | 2.540 |
| Ttc39c | NM_001077231 | tetratricopeptide repeat domain 39C | 2.548 |
| RGD1307461 | NM_001106854 | similar to RIKEN cDNA 6430571L13 gene; similar to g20 protein | 2.553 |
| Vcan | NM_001170558, NM_001170559, NM_001170560, NM_053663 | versican | 2.557 |
| Mir149 | NR_128668 | microRNA 149 | 2.581 |
| Plekhn1 | NM_001134523 | pleckstrin homology domain containing N1 | 2.583 |
| Kif26a | NM_001170348 | kinesin family member 26A | 2.598 |
| Bgn | NM_017087 | biglycan | 2.599 |
| Sox11 | NM_053349 | SRY box 11 | 2.609 |
| Gal | NM_033237 | galanin and GMAP prepropeptide | 2.612 |
| Kdm6b | NM_001108829 | lysine demethylase 6B | 2.618 |
| Igsf9 | NM_001107197 | immunoglobulin superfamily, member 9 | 2.621 |
| Scin | NM_198748 | scinderin | 2.622 |
| Gcnt1 | NM_022276 | glucosaminyl (N-acetyl) transferase 1, core 2 | 2.633 |
| Nrip3 | NM_001108498 | nuclear receptor interacting protein 3 | 2.634 |
| Pxdn | NM_001271261 | peroxidasin homolog (Drosophila) | 2.636 |
| Pbx1 | NM_001100681, NM_001134862 | pre-B-cell leukemia homeobox 1 | 2.639 |
| Hes3 | NM_022687 | hes family bHLH transcription factor 3 | 2.642 |
| Sox4 | NM_001271205 | SRY box 4 | 2.649 |
| Galnt10 | NM_130742 | polypeptide N-acetylgalactosaminyltransferase 10 | 2.662 |
| Per1 | NM_001034125 | period circadian clock 1 | 2.688 |
| Btg1 | NM_017258 | B-cell translocation gene 1, anti-proliferative | 2.698 |
| Synpr | NM_023974 | synaptoporin | 2.704 |
| Maff | NM_001130573 | MAF bZIP transcription factor F | 2.708 |
| Junb | NM_021836 | JunB proto-oncogene, AP-1 transcription factor subunit | 2.709 |
| Mir3597-2 | NR_037359 | microRNA 3597-2 | 2.715 |
| Samsn1 | NM_130821 | SAM domain, SH3 domain and nuclear localization signals, 1 | 2.723 |
| Isoc1 | NM_001014242 | isochorismatase domain containing 1 | 2.726 |
| Otop2 | NM_001105851 | otopetrin 2 | 2.727 |
| Hbb-b1 | NM_198776 | hemoglobin, beta adult major chain | 2.752 |
| Igfbp2 | NM_013122 | insulin-like growth factor binding protein 2 | 2.765 |
| RT1-N1 | NM_012646 | RT1 class Ib, locus N1 | 2.768 |
| Adamts19 | NM_001108433 | ADAM metallopeptidase with thrombospondin type 1 motif, 19 | 2.794 |
| Vav2 | NM_001106563 | vav guanine nucleotide exchange factor 2 | 2.804 |
| Mirlet7d | NR_031774 | microRNA let-7d | 2.814 |
| Mt2A | NM_001137564 | metallothionein 2A | 2.815 |
| Mfap2 | NM_001107989 | microfibrillar-associated protein 2 | 2.816 |
| Arx | NM_001100174 | aristaless related homeobox | 2.820 |
| Pgc | NM_133284 | progastricsin | 2.831 |
| Mir3596b | NR_037375 | microRNA 3596b | 2.840 |
| Syt10 | NM_031666 | synaptotagmin 10 | 2.841 |
| Steap2 | NM_001107846 | STEAP2 metalloreductase | 2.847 |
| Qprt | NM_001009646 | quinolinate phosphoribosyltransferase | 2.860 |
| Sall3 | NM_001108892 | spalt-like transcription factor 3 | 2.875 |
| Cartpt | NM_017110 | CART prepropeptide | 2.935 |
| Shisa3 | NM_001109087 | shisa family member 3 | 2.947 |
| Gsap | NM_001107845 | gamma-secretase activating protein | 2.960 |
| RT1-CE10 | NM_001008833 | RT1 class I, locus CE10 | 3.013 |
| Cxcl10 | NM_139089 | C-X-C motif chemokine ligand 10 | 3.014 |
| Rasa2 | NM_001105724 | RAS p21 protein activator 2 | 3.015 |
| Nnat | NM_001270867, NM_053601, NM_181687, NR_073089 | neuronatin | 3.017 |
| Aqp1 | NM_012778 | aquaporin 1 | 3.019 |
| Mx1 | NM_001271058, NM_001271059, NM_001271060, NM_001271061, NM_001271062, NM_173096 | myxovirus (influenza virus) resistance 1 | 3.020 |
| Kank3 | NM_001108989 | KN motif and ankyrin repeat domains 3 | 3.027 |
| Slc6a11 | NM_024372 | solute carrier family 6 member 11 | 3.042 |
| Mgst1 | NM_134349 | microsomal glutathione S-transferase 1 | 3.052 |
| Fmo1 | NM_012792 | flavin containing monooxygenase 1 | 3.054 |
| Mtmr11 | NM_001191096 | myotubularin related protein 11 | 3.064 |
| Cacng5 | NM_080693 | calcium voltage-gated channel auxiliary subunit gamma 5 | 3.075 |
| Reck | NM_001107954 | reversion-inducing-cysteine-rich protein with kazal motifs | 3.090 |
| Ablim3 | NM_001191698 | actin binding LIM protein family, member 3 | 3.100 |
| LOC100361087 | NM_001190459 | hypothetical LOC100361087 | 3.101 |
| C1ql2 | NM_001105949 | complement component 1, q subcomponent-like 2 | 3.107 |
| Slc43a3 | NM_001107743 | solute carrier family 43, member 3 | 3.118 |
| RGD1564664 | NM_001110055 | similar to LOC387763 protein | 3.135 |
| LOC100134871 | NM_001113223 | beta globin minor gene | 3.136 |
| Sfrp1 | NM_001276712 | secreted frizzled-related protein 1 | 3.155 |
| Fos | NM_022197 | FBJ osteosarcoma oncogene | 3.157 |
| Mir3546 | NR_037320 | microRNA 3546 | 3.199 |
| Ppm1j | NM_001005540 | protein phosphatase, Mg2+/Mn2+ dependent, 1J | 3.209 |
| Grip2 | NM_138535 | glutamate receptor interacting protein 2 | 3.226 |
| Hmgcs2 | NM_173094 | 3-hydroxy-3-methylglutaryl-CoA synthase 2 | 3.227 |
| Cdca7l | NM_001034953 | cell division cycle associated 7 like | 3.235 |
| Cyp39a1 | NM_001106893 | cytochrome P450, family 39, subfamily a, polypeptide 1 | 3.252 |
| Nr4a1 | NM_024388 | nuclear receptor subfamily 4, group A, member 1 | 3.269 |
| Tuba8 | NM_001024339 | tubulin, alpha 8 | 3.276 |
| Cpne6 | NM_001191113 | copine VI (neuronal) | 3.284 |
| Ap1s2 | NM_001127531 | adaptor-related protein complex 1, sigma 2 subunit | 3.288 |
| Eml5 | NM_001003402 | echinoderm microtubule associated protein like 5 | 3.297 |
| Svil | NM_001108416 | supervillin | 3.352 |
| Ampd2 | NM_001101681 | adenosine monophosphate deaminase 2 | 3.354 |
| Dusp5 | NM_133578 | dual specificity phosphatase 5 | 3.372 |
| Slc17a6 | NM_053427 | solute carrier family 17 member 6 | 3.414 |
| Pcdha4 | NM_053933 | protocadherin alpha 4 | 3.442 |
| Mirlet7c-2 | NR_031804 | microRNA let7c-2 | 3.455 |
| Rnd3 | NM_001007641 | Rho family GTPase 3 | 3.463 |
| Zfp385d | NM_001013992 | zinc finger protein 385D | 3.464 |
| C3 | NM_016994 | complement component 3 | 3.488 |
| Map3k1 | NM_053887 | mitogen-activated protein kinase kinase kinase 1 | 3.527 |
| Meis2 | NM_001107758 | Meis homeobox 2 | 3.578 |
| Gipr | NM_012714 | gastric inhibitory polypeptide receptor | 3.591 |
| Col3a1 | NM_032085 | collagen type III alpha 1 chain | 3.614 |
| Ptpro | NM_017336 | protein tyrosine phosphatase, receptor type, O | 3.614 |
| Amigo2 | NM_182816 | adhesion molecule with Ig like domain 2 | 3.635 |
| Prokr2 | NM_138978 | prokineticin receptor 2 | 3.637 |
| Vash2 | NM_001109082 | vasohibin 2 | 3.669 |
| Kcnj16 | NM_053314 | potassium voltage-gated channel subfamily J member 16 | 3.675 |
| Fgl2 | NM_053455 | fibrinogen-like 2 | 3.702 |
| Tlk1 | NM_001107734 | tousled-like kinase 1 | 3.754 |
| Pax6 | NM_013001 | paired box 6 | 3.754 |
| Ppp1r17 | NM_153467 | protein phosphatase 1, regulatory subunit 17 | 3.761 |
| Igfbp5 | NM_012817 | insulin-like growth factor binding protein 5 | 3.762 |
| Gnb3 | NM_021858 | G protein subunit beta 3 | 3.812 |
| Inpp5j | NM_133562 | inositol polyphosphate-5-phosphatase J | 3.834 |
| Alx3 | NM_001007012 | ALX homeobox 3 | 3.844 |
| Vipr2 | NM_017238 | vasoactive intestinal peptide receptor 2 | 3.875 |
| Crabp1 | NM_001105716 | cellular retinoic acid binding protein 1 | 3.876 |
| Acbd7 | NM_001126079 | acyl-CoA binding domain containing 7 | 3.892 |
| Syt6 | NM_022191 | synaptotagmin 6 | 3.902 |
| Slc35d3 | NM_001107522 | solute carrier family 35, member D3 | 3.922 |
| Tbx21 | NM_001107043 | T-box 21 | 3.976 |
| Cdca7 | NM_001025693 | cell division cycle associated 7 | 4.031 |
| Kcnab3 | NM_031652 | potassium voltage-gated channel subfamily A regulatory beta subunit 3 | 4.048 |
| Tnc | NM_053861 | tenascin C | 4.067 |
| Fabp7 | NM_030832 | fatty acid binding protein 7 | 4.089 |
| Krt71 | NM_001276440 | keratin 71, type II | 4.106 |
| Gad1 | NM_017007 | glutamate decarboxylase 1 | 4.116 |
| Gchfr | NM_133595 | GTP cyclohydrolase I feedback regulator | 4.153 |
| Ctxn3 | NM_001134696 | cortexin 3 | 4.347 |
| Wnt5a | NM_022631 | wingless-type MMTV integration site family, member 5A | 4.479 |
| Fosb | NM_001256509 | FBJ osteosarcoma oncogene B | 4.549 |
| Rpp25 | NM_001012124 | ribonuclease P/MRP 25 subunit | 4.605 |
| Crhr2 | NM_022714 | corticotropin releasing hormone receptor 2 | 4.681 |
| Mirlet7b | NR_031802 | microRNA let-7b | 4.783 |
| Mlf1 | NM_001107680 | myeloid leukemia factor 1 | 4.793 |
| Sall1 | NM_001107415 | spalt-like transcription factor 1 | 4.881 |
| Myo16 | NM_138893 | myosin XVI | 4.891 |
| Csdc2 | NM_001170542 | cold shock domain containing C2 | 4.960 |
| Sphk1 | NM_001270807, NM_001270808, NM_001270809, NM_001270810, NM_001270811, NM_133386 | sphingosine kinase 1 | 5.107 |
| Meis1 | NM_001134702 | Meis homeobox 1 | 5.200 |
| Klhl14 | NM_001108885 | kelch-like family member 14 | 5.247 |
| Slc17a4 | NM_001271214 | solute carrier family 17, member 4 | 5.249 |
| Ly6g6d | NM_001001970 | lymphocyte antigen 6 complex, locus G6D | 5.263 |
| Pcp4l1 | NM_001126093 | Purkinje cell protein 4-like 1 | 5.389 |
| Lrrc23 | NM_001013165 | leucine rich repeat containing 23 | 5.394 |
| Lgr5 | NM_001106784 | leucine rich repeat containing G protein coupled receptor 5 | 5.463 |
| Pcbp3 | NM_001011945 | poly(rC) binding protein 3 | 5.763 |
| Trdn | NM_021666 | triadin | 5.873 |
| Cdhr1 | NM_053572 | cadherin-related family member 1 | 5.906 |
| Dlx5 | NM_012943 | distal-less homeobox 5 | 6.033 |
| Doc2g | NM_001011937 | double C2-like domains, gamma | 6.051 |
| Dlx1 | NM_001100531 | distal-less homeobox 1 | 6.128 |
| Sp7 | NM_001037632, NM_181374 | Sp7 transcription factor | 6.254 |
| Igfbpl1 | NM_001108972 | insulin-like growth factor binding protein-like 1 | 6.258 |
| Dcx | NM_053379 | doublecortin | 6.274 |
| Mrap | NM_001135834 | melanocortin 2 receptor accessory protein | 6.403 |
| Dlx2 | NM_001191746 | distal-less homeobox 2 | 6.614 |
| Car3 | NM_019292 | carbonic anhydrase 3 | 6.789 |
| Gpsm1 | NM_001145469, NM_144745 | G-protein signaling modulator 1 | 7.013 |
| Sp9 | NM_001191902 | trans-acting transcription factor 9 | 7.145 |
| Th | NM_012740 | tyrosine hydroxylase | 7.814 |
| Itga10 | NM_001107699 | integrin subunit alpha 10 | 7.914 |
| Nrl | NM_001106036 | neural retina leucine zipper | 8.109 |
| Ly6g6e | NM_001001972 | lymphocyte antigen 6 complex, locus G6E | 8.355 |
| Fcrl2 | NM_001107702 | Fc receptor-like 2 | 8.385 |
| Nmb | NM_001109149 | neuromedin B | 9.152 |
| Calb2 | NM_053988 | calbindin 2 | 9.395 |
| Ms4a15 | NM_001191897 | membrane spanning 4-domains A15 | 10.519 |
| Eya2 | NM_130427 | EYA transcriptional coactivator and phosphatase 2 | 11.666 |
| Myl6l | NM_001100983 | myosin, light polypeptide 6, alkali, smooth muscle and non-muscle-like | 11.712 |
| Pbx3 | NM_001107834 | PBX homeobox 3 | 11.842 |
| Trh | NM_013046 | thyrotropin releasing hormone | 12.380 |
| Sp8 | NM_001191774 | Sp8 transcription factor | 13.195 |
| Shisa8 | NM_001207022 | shisa family member 8 | 13.470 |
| Scgn | NM_201561 | secretagogin, EF-hand calcium binding protein | 17.395 |
| Omp | NM_012616 | olfactory marker protein | 21.206 |
| Spp1 | NM_012881 | secreted phosphoprotein 1 | 25.615 |
| S100a5 | NM_001106438 | S100 calcium binding protein A5 | 56.223 |

FC, fold change

**Table S5.** Gene-enrichment analysis on differentially expressed genes (DEGs) by sleep deprivation (SD)

| KEGG MapID | Map Name | No of significant genes | ­*p*-value | FDR *p*-values |
| --- | --- | --- | --- | --- |
| 04080 | Neuroactive ligand-receptor interaction | 22 | <0.001 | <0.001 |
| 04020 | Calcium signaling pathway | 14 | <0.001 | <0.001 |
| 05031 | Amphetamine addiction | 10 | <0.001 | <0.001 |
| 04024 | cAMP signaling pathway | 14 | <0.001 | <0.001 |
| 05030 | Cocaine addiction | 9 | <0.001 | <0.001 |
| 01100 | Metabolic pathways | 31 | <0.001 | <0.001 |
| 04360 | Axon guidance | 12 | <0.001 | <0.001 |
| 05165 | Human papillomavirus infection | 15 | <0.001 | <0.001 |
| 05034 | Alcoholism | 11 | <0.001 | <0.001 |
| 05202 | Transcriptional misregulation in cancer | 10 | <0.001 | <0.001 |
| 04514 | Cell adhesion molecules (CAMs) | 10 | <0.001 | <0.001 |
| 05166 | HTLV-I infection | 12 | <0.001 | <0.001 |
| 04145 | Phagosome | 10 | <0.001 | <0.001 |
| 05012 | Parkinson's disease | 9 | <0.001 | <0.001 |
| 04971 | Gastric acid secretion | 7 | <0.001 | <0.001 |
| 05200 | Pathways in cancer | 13 | <0.001 | <0.001 |
| 04512 | ECM-receptor interaction | 7 | <0.001 | 0.001 |
| 04728 | Dopaminergic synapse | 8 | <0.001 | 0.001 |
| 04371 | Apelin signaling pathway | 8 | <0.001 | 0.001 |
| 04261 | Adrenergic signaling in cardiomyocytes | 8 | <0.001 | 0.001 |
| 05144 | Malaria | 6 | <0.001 | 0.001 |
| 04713 | Circadian entrainment | 7 | <0.001 | 0.001 |
| 04721 | Synaptic vesicle cycle | 6 | <0.001 | 0.001 |
| 05206 | MicroRNAs in cancer | 10 | <0.001 | 0.001 |
| 04810 | Regulation of actin cytoskeleton | 9 | <0.001 | 0.002 |
| 05133 | Pertussis | 6 | <0.001 | 0.002 |
| 04970 | Salivary secretion | 6 | <0.001 | 0.002 |
| 04726 | Serotonergic synapse | 7 | <0.001 | 0.002 |
| 04260 | Cardiac muscle contraction | 6 | <0.001 | 0.002 |
| 04610 | Complement and coagulation cascades | 6 | <0.001 | 0.003 |
| 04550 | Signaling pathways regulating pluripotency of stem cells | 7 | <0.001 | 0.004 |
| 05167 | Kaposi's sarcoma-associated herpesvirus infection | 8 | <0.001 | 0.004 |
| 04310 | Wnt signaling pathway | 7 | <0.001 | 0.004 |
| 05224 | Breast cancer | 7 | <0.001 | 0.004 |
| 04640 | Hematopoietic cell lineage | 6 | <0.001 | 0.004 |
| 05205 | Proteoglycans in cancer | 8 | <0.001 | 0.004 |
| 04972 | Pancreatic secretion | 6 | <0.001 | 0.004 |
| 05150 | Staphylococcus aureus infection | 5 | <0.001 | 0.005 |
| 04612 | Antigen processing and presentation | 6 | <0.001 | 0.005 |
| 05168 | Herpes simplex infection | 8 | <0.001 | 0.005 |
| 04724 | Glutamatergic synapse | 6 | 0.001 | 0.008 |
| 05322 | Systemic lupus erythematosus | 6 | 0.002 | 0.012 |
| 04218 | Cellular senescence | 7 | 0.002 | 0.012 |
| 04010 | MAPK signaling pathway | 8 | 0.002 | 0.012 |
| 04940 | Type I diabetes mellitus | 5 | 0.003 | 0.013 |
| 05143 | African trypanosomiasis | 4 | 0.003 | 0.015 |
| 04911 | Insulin secretion | 5 | 0.003 | 0.017 |
| 04727 | GABAergic synapse | 5 | 0.004 | 0.02 |
| 04974 | Protein digestion and absorption | 5 | 0.004 | 0.021 |
| 04978 | Mineral absorption | 4 | 0.004 | 0.021 |
| 04390 | Hippo signaling pathway | 6 | 0.005 | 0.023 |
| 05203 | Viral carcinogenesis | 7 | 0.006 | 0.025 |
| 05217 | Basal cell carcinoma | 4 | 0.007 | 0.032 |
| 04270 | Vascular smooth muscle contraction | 5 | 0.011 | 0.049 |
| 04964 | Proximal tubule bicarbonate reclamation | 3 | 0.011 | 0.049 |

Gene-enrichment analysis for DEGs was performed based on the KEGG pathway database (https://www.genome.jp/kegg/). The ­*p*-values were calculated based on a modified Fisher’s exact test. The false discovery rate (FDR) was controlled by adjusting the *p*-value using the Benjamini-Hochberg algorithm.

**Table S6.** Expressions of neurotransmitter synapse pathway genes in the prefrontal cortex of sleep deprived (SD) rats

| Map ID | Map Name | Transcript ID | Gene | Description | FC | FDR  p-value |
| --- | --- | --- | --- | --- | --- | --- |
|  |  |  |  |  | SD/Con |  |
| 04728 | Dopaminergic synapse | NM_138521 | Ppp1r1b | protein phosphatase 1, regulatory (inhibitor) subunit 1B | -3.424 | 0.001 |
|  |  | NM_022197 | Fos | FBJ osteosarcoma oncogene | 3.157 |  |
|  |  | NM_001007235,  NM_001270596,  NM_001270597 | Itpr1 | inositol 1,4,5-trisphosphate receptor, type 1 | -2.059 |  |
|  |  | NM_012740 | Th | tyrosine hydroxylase | 7.814 |  |
|  |  | NM_021858 | Gnb3 | G protein subunit beta 3 | 3.812 |  |
|  |  | NM_012546 | Drd1 | dopamine receptor D1 | -2.724 |  |
|  |  | NM_012547 | Drd2 | dopamine receptor D2 | -2.431 |  |
|  |  | NM_012694 | Slc6a3 | solute carrier family 6 member 3 | 2.129 |  |
| 04726 | Serotonergic synapse | NM_001007235,  NM_001270596,  NM_001270597 | Itpr1 | inositol 1,4,5-trisphosphate receptor, type 1 | -2.059 | 0.002 |
|  |  | NM_021858 | Gnb3 | G protein subunit beta 3 | 3.812 |  |
|  |  | NM_031010 | Alox15 | arachidonate 15-lipoxygenase | 2.078 |  |
|  |  | NM_021857 | Htr1f | 5-hydroxytryptamine receptor 1F | 2.107 |  |
|  |  | NM_024365 | Htr6 | 5-hydroxytryptamine receptor 6 | -2.673 |  |
|  |  | NM_024395 | Htr5b | 5-hydroxytryptamine (serotonin) receptor 5B | 2.046 |  |
|  |  | NM_153301 | Alox15b | arachidonate 15-lipoxygenase, type B | 2.042 |  |
| 04724 | Glutamatergic synapse | NM_001007235,  NM_001270596,  NM_001270597 | Itpr1 | inositol 1,4,5-trisphosphate receptor, type 1 | -2.059 | 0.008 |
|  |  | NM_022666 | Grm4 | glutamate metabotropic receptor 4 | 2.267 |  |
|  |  | NM_053309 | Homer2 | homer scaffolding protein 2 | 2.009 |  |
|  |  | NM_053427 | Slc17a6 | solute carrier family 17 member 6 | 3.414 |  |
|  |  | NM_021858 | Gnb3 | G protein subunit beta 3 | 3.812 |  |
|  |  | NM_133308 | Grin3b | glutamate ionotropic receptor NMDA type subunit 3B | 2.540 |  |
| 04727 | GABAergic synapse | NM_017007 | Gad1 | glutamate decarboxylase 1 | 4.116 | 0.020 |
|  |  | NM_024133,  NM_177982 | Hap1 | huntingtin-associated protein 1 | 2.225 |  |
|  |  | NM_031782 | Slc32a1 | solute carrier family 32 member 1 | 2.418 |  |
|  |  | NM_024372 | Slc6a11 | solute carrier family 6 member 11 | 3.042 |  |
|  |  | NM_021858 | Gnb3 | G protein subunit beta 3 | 3.812 |  |

FC, fold change

**miRNA-Seq.** The next-generation sequencing was performed using the RNA pooled from 4 rats per group. The total RNA was isolated from the prefrontal cortex samples pooled in each group using TRIzol. RNA integrity was measured using an Agilent 2100 Bioanalyzer (Agilent Technologies, Inc., Santa Clara, CA, USA). The cDNA libraries were constructed using the TruSeq Small RNA Library kit on 1 μg of total RNA according to the following process: fragmentation of RNA, reverse transcription, and sequencing using Illumina HiSeq2500 (library size is ranged from 145 bp to 160 bp). The low quality and adapter sequences were eliminated from the raw reads of the sequencer. And then, the reads were aligned to the Rattus norvegicus based on miRBase v21 (https://www.mirbase.org/) using Bowtie 2. The aligned reads were assembled into the transcripts, and their abundance was estimated using miRDeep2 v2.0.0.7. The relative abundance was estimated as reads per million (RPM) of mature miRNAs expressed in each group.

**Statistical Analysis of Gene Expression Level.** To select differentially expressed miRNAs (DEmiRNAs), the relative abundances for each miRNA were compared between groups. miRNAs whose RPM value was 0 in any of the groups were excluded from the analysis. After 1 was added to the RPM values of mature miRNAs, the values were converted based on log2 and then were subjected to quantile normalization. We determined the differential expression data by ∣fold change (FC)∣ ≥ 2 and independent t-test. To measure the similarity, hierarchical clustering for the DEmiRNA set was conducted using complete linkage and Euclidean distance. All data analysis on miRNA-seq was conducted using R 3.4.3 (<http://www.r-project.org/>).

**Altered miRNA Expression in the Prefrontal Cortex of SD Rats.** Through RNA-seq, we obtained 67,782,419 and 57,424,761 raw reads from the control and SD groups, respectively. After eliminating low-quality reads and adaptor sequences, we finally 44,288,093 from control group, and 43,735,930 reads from SD group. The length distribution of most of the clean reads was 20-25 nt, which is the typical size of mature miRNAs (Figure S1). These clean reads were then aligned to the Rattus norvegicus genome using Bowtie software. We found 25,208,098 (control) and 20,962,290 miRNA reads (SD), which accounted for 56.90%, and 47.90% of the total reads (filtered read), respectively (Table S7).

Finally, we acquired the expression data on 557 mature miRNAs. For the DEmiRNA analysis, miRNAs with ∣FC∣ ≥ 2.0 and the *p* value < 0.05 in the comparison between control and SD rats were selected. A total of 74 DEmiRNAs were identified in the control vs. SD comparison (Table S8).


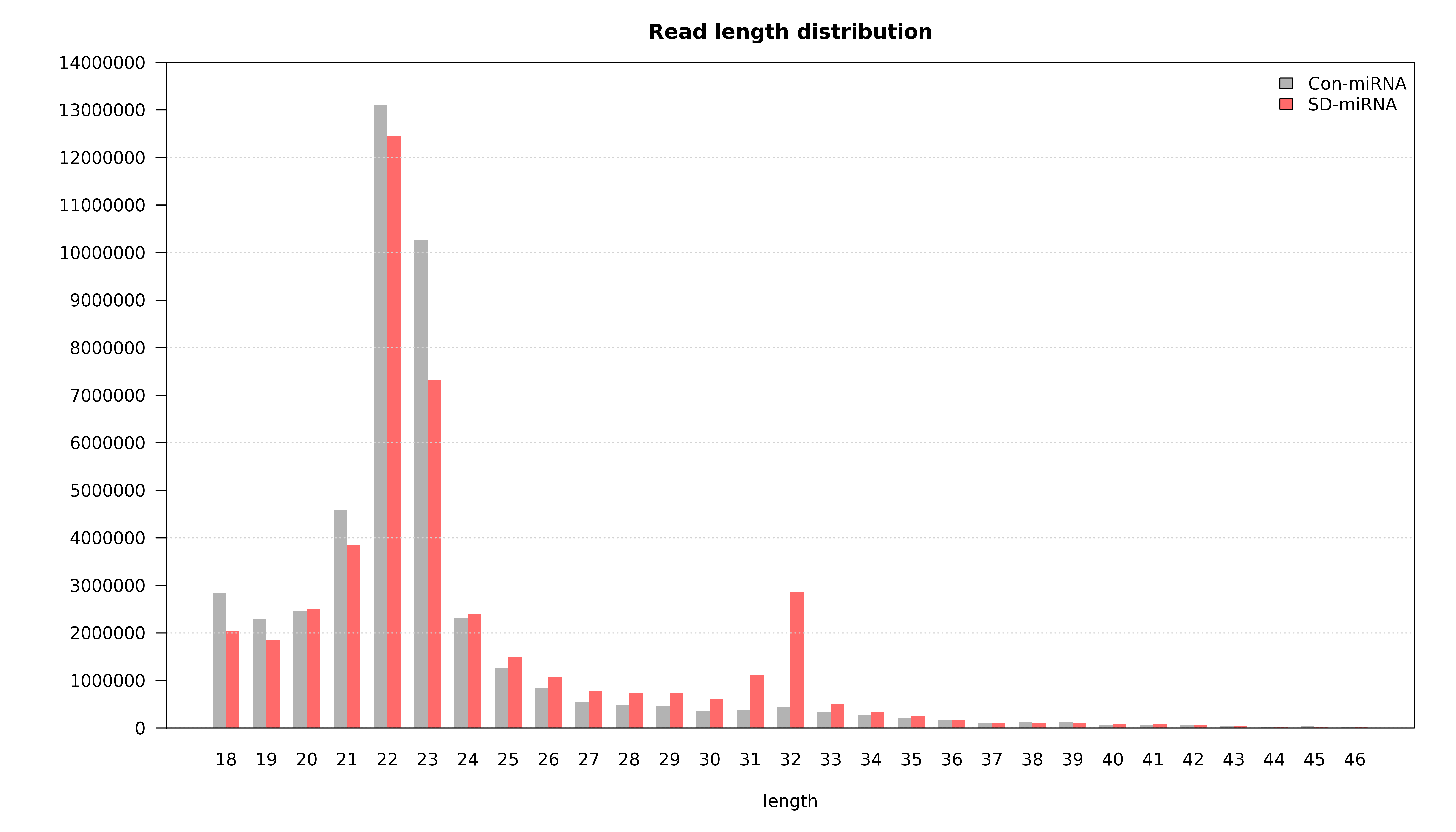


**Figure S1.** Read length distribution of trimmed reads

**Table S7.** Mapped reads to miRBase precursor in miRNA-seq analysis

| Sample name | Filtered total reads | Mapped reads | Unique clustered reads* | Mapped unique clustered reads |
| --- | --- | --- | --- | --- |
| Con | 44,288,093 | 25,208,098  (56.90%) | 3,495,306 | 23,826 |
| SD | 43,735,930 | 20,962,290  (47.90%) | 5,400,702 | 27,158 |

*This cluster contains reads that are 100% match to the sequence identity and read length.

**Table S8.** Altered expressions of mature microRNAs (miRNAs) in sleep deprivation (SD) rats.

| Mature | NCBI Gene ID | Fold change |
| --- | --- | --- |
|  |  | SD/Con |
| rno-miR-183-5p | 100314041 | 5.693 |
| rno-miR-200c-3p | 100314049 | 4.240 |
| rno-miR-29c-5p | 100314187 | 4.177 |
| rno-miR-200a-3p | 100314194 | 3.871 |
| rno-miR-200b-5p | 100314050 | 3.336 |
| rno-miR-200b-3p | 100314050 | 3.151 |
| rno-miR-331-3p | 100313975 | 3.113 |
| rno-miR-182 | 100314172 | 3.012 |
| rno-miR-495 | 100314111 | 2.963 |
| rno-miR-429 | 100314067 | 2.814 |
| rno-miR-200a-5p | 100314194 | 2.793 |
| rno-miR-3068-5p | - | 2.714 |
| rno-miR-129-2-3p | 100313984 | 2.672 |
| rno-miR-129-1-3p | 100314157 | 2.638 |
| rno-miR-141-3p | 100314215 | 2.576 |
| rno-miR-183-3p | 100314041 | 2.566 |
| rno-miR-29b-3p | 100314007 | 2.529 |
| rno-miR-455-3p | 100314205 | 2.435 |
| rno-miR-673-3p | 100314114 | 2.369 |
| rno-miR-24-3p | 100314003 | 2.264 |
| rno-miR-344a-3p | 100313981 | 2.229 |
| rno-miR-193b-3p | - | 2.220 |
| rno-miR-154-5p | 100314039 | 2.220 |
| rno-miR-466b-3p | 100314093 | 2.200 |
| rno-miR-139-5p | 100314240 | 2.197 |
| rno-miR-381-5p | 100314084 | 2.169 |
| rno-miR-598-3p | 100314206 | 2.157 |
| rno-miR-145-5p | 100314036 | 2.144 |
| rno-miR-133b-3p | 100314078 | 2.112 |
| rno-miR-324-5p | 100314181 | 2.106 |
| rno-miR-450b-3p | - | 2.105 |
| rno-miR-191a-5p | 100314045 | 2.082 |
| rno-miR-34a-5p | 100314015 | 2.066 |
| rno-miR-664-3p | 100314170 | 2.052 |
| rno-miR-124-3p | 100314155 | 2.016 |
| rno-miR-211-5p | 100314054 | -2.005 |
| rno-miR-431 | 100314165 | -2.016 |
| rno-miR-674-3p | 100314115 | -2.023 |
| rno-miR-326-3p | 100313971 | -2.024 |
| rno-miR-3550 | 100526620 | -2.049 |
| rno-miR-487b-3p | 100314284 | -2.050 |
| rno-miR-6314 | - | -2.054 |
| rno-miR-28-5p | 100314152 | -2.059 |
| rno-miR-3064-5p | - | -2.067 |
| rno-miR-370-3p | 100314254 | -2.092 |
| rno-miR-423-5p | 100314264 | -2.107 |
| rno-miR-935 | 100314272 | -2.163 |
| rno-miR-149-5p | - | -2.169 |
| rno-miR-667-3p | 100314139 | -2.242 |
| rno-miR-325-3p | 100313970 | -2.256 |
| rno-miR-3473 | - | -2.258 |
| rno-miR-877 | 100314097 | -2.315 |
| rno-miR-330-5p | 100313974 | -2.337 |
| rno-miR-195-3p | 100314281 | -2.347 |
| rno-miR-125a-5p | 100314236 | -2.380 |
| rno-miR-485-5p | 100314086 | -2.508 |
| rno-miR-3085 | 100526566 | -2.520 |
| rno-miR-1298 | - | -2.609 |
| rno-miR-1247-5p | - | -2.676 |
| rno-miR-3544 | 100526619 | -2.703 |
| rno-miR-540-3p | 100314255 | -2.731 |
| rno-miR-125b-1-3p | 100314156 | -2.823 |
| rno-miR-27b-5p | 100314005 | -2.875 |
| rno-miR-30c-1-3p | 100314009 | -2.969 |
| rno-miR-150-5p | 100314158 | -3.035 |
| rno-miR-383-5p | 100314072 | -3.157 |
| rno-miR-298-5p | 100314250 | -3.236 |
| rno-miR-210-5p | 100314053 | -3.238 |
| rno-miR-764-5p | 100314274 | -3.493 |
| rno-miR-423-3p | 100314264 | -3.637 |
| rno-miR-671 | 100314113 | -3.820 |
| rno-miR-92b-3p | 100314119 | -4.252 |
| rno-miR-3552 | 100526569 | -5.412 |
| rno-miR-3577 | 100526638 | -5.552 |

**Selection of predicted target miRNAs on Th gene.** Among 55 significant pathways detected through the enrichment analysis We focused on the dopaminergic synapse pathway. A number of neurotransmitters have been implicated in the pathology of mania, including dopamine, serotonin, norepinephrine and γ-aminobutyric acid (GABA). Since the 1970s, dopamine has been first reported to be involved in the pathophysiology of mania, and dysregulation of dopaminergic neurotransmission have been consistently reported in BPD.^1-5^ In addition, our enrichment analysis shows lower *p*-value in dopaminergic synapse pathway (FDR-corrected *p* = 0.001) than any other neurotransmitter synapse pathways (Table S5). SD altered expressions of 8 genes belonging to the dopamine synapse pathway (Table S6). In particular, Th, Slc6a3, Drd1 and Drd2, which play a key role in dopaminergic transmission such as dopamine synthesis, dopamine reuptake, and production of biological response by dopamine, were included.

Target miRNAs on dopamine synapse genes were predicted by the TargetScan v8.0 database (<https://www.targetscan.org/vert_80/>) among 74 DEmiRNAs. Because miRNAs promote mRNA degradation and repression of mRNA translation, binding to their target sites on 3’UTR of genes,^6^ decreased miRNAs were selected on genes with increased expression levels, and increased miRNAs were selected on genes with decreased expression levels. Table S4 showed dopamine synapse genes altered in the prefrontal cortex of SD rats and predicted target miRNAs on the genes. Particularly, we focused on Th whose mRNA expression was most markedly increased among the dopamine synapse genes. TH is an enzyme responsible for catalyzing the conversion of L-tyrosine to L-DOPA, which is the rate limiting step in dopamine synthesis. An increased release of dopamine in axonal terminal induces the elevation of the mRNA and protein expression of TH.^7^

**Table S9.** Dopamine synapse genes altered by sleep deprivation (SD) and predicted target microRNAs (miRNAs) on the dopamine synapse genes.

| Gene  /Transcript ID | Description | FC | miRNA | FC | Position in the 3'UTR | Seed match^a^ | weighted context++ score^b^ |
| --- | --- | --- | --- | --- | --- | --- | --- |
|  |  | SD/Con |  | SD/Con |  |  |  |
| TH  NM_012740 | tyrosine hydroxylase | 7.814 | rno-miR-330-5p | -2.337 | 74-80 | 7mer-m8 | -0.11 |
|  |  |  | rno-miR-326-3p | -2.024 | 74-80 | 7mer-m8 | -0.09 |
|  |  |  | rno-miR-325-3p | -1.256 | 227-234 | 8mer | -0.21 |
| Gnb3  NM_021858 | G protein subunit beta 3 | 3.812 | rno-miR-150-5p | -3.035 | 144-150 | 7mer-m8 | -0.12 |
|  |  |  | rno-miR-667-3p | -2.242 | 329-336 | 8mer | -0.52 |
| Fos  NM_022197 | FBJ osteosarcoma oncogene | 3.157 | rno-miR-1298 | -2.609 | 765-771 | 7mer-1A | 0 |
|  |  |  | rno-miR-149-5p | -2.169 | 307-313 | 7mer-m8 | -0.23 |
|  |  |  | rno-miR-935 | -2.163 | 594-601 | 8mer | -0.36 |
|  |  |  | rno-miR-431 | -2.016 | 802-808 | 7mer-1A | 0 |
|  |  |  | rno-miR-211-5p | -2.005 | 1154-1160 | 7mer-m8 | 0 |
|  |  |  | rno-miR-211-5p | -2.005 | 1220-1226 | 7mer-1A | 0 |
| Slc6a3  NM_012694 | solute carrier family 6 member 3 | 2.129 | rno-miR-3577 | -5.552 | 38-44 | 7mer-m8 | -0.1 |
|  |  |  | rno-miR-150-5p | -3.035 | 318-324 | 7mer-1A | -0.01 |
|  |  |  | rno-miR-423-3p | -3.637 | 514-520 | 7mer-1A | -0.19 |
|  |  |  | rno-miR-210-5p | -3.238 | 1077-1083 | 7mer-1A | -0.15 |
|  |  |  | rno-miR-298-5p | -3.236 | 1112-1118 | 7mer-1A | -0.11 |
| Itpr1  NM_001007235,  NM_001270596,  NM_001270597 | inositol 1,4,5-trisphosphate receptor, type 1 | -2.059 | rno-miR-200c-3p | 4.24 | 332-338 | 7mer-m8 | -0.1 |
|  |  |  | rno-miR-200a-3p | 3.871 | 1323-1329 | 7mer-m8 | 0 |
|  |  |  | rno-miR-200b-3p | 3.151 | 332-338 | 7mer-m8 | -0.1 |
|  |  |  | rno-miR-331-3p | 3.113 | 1443-1449 | 7mer-m8 | 0 |
|  |  |  | rno-miR-182 | 3.012 | 211-217 | 7mer-1A | -0.13 |
|  |  |  | rno-miR-182 | 3.012 | 484-490 | 7mer-1A | -0.11 |
|  |  |  | rno-miR-429 | 2.814 | 332-338 | 7mer-m8 | -0.1 |
|  |  |  | rno-miR-3068-5p | 2.714 | 602-608 | 7mer-m8 | -0.06 |
|  |  |  | rno-miR-141-3p | 2.576 | 1323-1329 | 7mer-m8 | 0 |
|  |  |  | rno-miR-455-3p | 2.435 | 1348-1354 | 7mer-1A | 0 |
|  |  |  | rno-miR-193b-3p | 2.22 | 330-336 | 7mer-1A | -0.19 |
|  |  |  | rno-miR-466b-3p | 2.2 | 132-138 | 7mer-m8 | -0.14 |
|  |  |  | rno-miR-466b-3p | 2.2 | 446-452 | 7mer-1A | -0.02 |
|  |  |  | rno-miR-324-5p | 2.106 | 989-995 | 7mer-1A | -0.06 |
| Drd2  NM_012547 | dopamine receptor D2 | -2.431 | rno-miR-200a-3p | 3.871 | 127-134 | 8mer | -0.08 |
|  |  |  | rno-miR-141-3p | 2.576 | 127-134 | 8mer | -0.08 |
|  |  |  | rno-miR-3068-5p | 2.714 | 559-565 | 7mer-1A | -0.07 |
|  |  |  | rno-miR-193b-3p | 2.22 | 789-795 | 7mer-m8 | -0.16 |
|  |  |  | rno-miR-141-3p | 2.576 | 808-814 | 7mer-m8 | -0.02 |
|  |  |  | rno-miR-200a-3p | 3.871 | 808-814 | 7mer-m8 | -0.02 |
|  |  |  | rno-miR-124-3p | 2.016 | 901-907 | 7mer-1A | -0.06 |
| Drd1  NM_012546 | dopamine receptor D1 | -2.724 | rno-miR-429 | 2.814 | 171-177 | 7mer-1A | -0.01 |
|  |  |  | rno-miR-200c-3p | 4.24 | 171-177 | 7mer-1A | -0.01 |
|  |  |  | rno-miR-200b-3p | 3.151 | 171-177 | 7mer-1A | -0.01 |
|  |  |  | rno-miR-29b-3p | 2.529 | 288-294 | 7mer-1A | -0.22 |
|  |  |  | rno-miR-495 | 2.963 | 339-345 | 7mer-1A | -0.01 |
|  |  |  | rno-miR-664-3p | 2.052 | 457-463 | 7mer-1A | -0.03 |
|  |  |  | rno-miR-24-3p | 2.264 | 561-567 | 7mer-1A | -0.12 |
|  |  |  | rno-miR-466b-3p | 2.2 | 829-835 | 7mer-1A | -0.01 |
|  |  |  | rno-miR-466b-3p | 2.2 | 911-917 | 7mer-1A | -0.04 |
|  |  |  | rno-miR-455-3p | 2.435 | 1357-1363 | 7mer-m8 | -0.11 |
|  |  |  | rno-miR-200a-3p | 3.871 | 1405-1411 | 7mer-m8 | -0.18 |
|  |  |  | rno-miR-141-3p | 2.576 | 1405-1411 | 7mer-m8 | -0.18 |
| Ppp1r1b  NM_138521 | protein phosphatase 1, regulatory (inhibitor) subunit 1B | -3.424 | rno-miR-455-3p | 2.435 | 505-511 | 7mer-m8 | -0.09 |
|  |  |  | rno-miR-145-5p | 2.144 | 226-232 | 7mer-1A | -0.01 |
|  |  |  | rno-miR-450b-3p | 2.105 | 156-162 | 7mer-m8 | -0.24 |
|  |  |  | rno-miR-450b-3p | 2.105 | 389-395 | 7mer-m8 | -0.15 |

FC, fold change

Target miRNAs on genes were predicted by the TargetScan v8.0 database (<https://www.targetscan.org/vert_80/>). On increased genes, decreased miRNAs were selected, and increased miRNAs were selected on decreased genes.

^a^The matching sites in the seed region (nucleotides 2 to 8 from 5’ of miRNA that have perfect Watson-Crick pairing with the 3’ UTR), from the strictest to the least strict (the site efficacy, 8mer > 7mer > 7mer-A1 > 6mer).^8^

^b^Range of weighted context++ score is from1 to -1. The scores with a lower negative value indicate a greater prediction of repression.^8^

**Table S10.** Primer sequences for quantitative real-time PCR

| Gene | Primers |  |
| --- | --- | --- |
| Primers for mRNA analysis | | |
| Drd1 | Forward: | 5'-CAGTATGAGAGGAAGATG-3' |
|  | Reverse: | 5'-AGGATATAAGGACAGACA-3' |
| Drd2 | Forward: | 5'-AACAATACAGACCAGAATG-3' |
|  | Reverse: | 5'-TGATATAGACCAGCAGAG-3' |
| Slc6a3 | Forward: | 5'-GAGTGCTGATTGCCTTCT-3' |
|  | Reverse: | 5'-ATGGAGGTGGTGATGATTG-3' |
| Th | Forward: | 5'-AGAGATTGCCTTCCAGTA-3' |
|  | Reverse: | 5'-TGACATATACCTCCTTCCA-3' |
| Ubc | Forward: | 5'-AGAAGGTCAAACAGGAAGATA-3' |
|  | Reverse: | 5'-CCAAGAACAAGCACAAGAA-3' |
| Primers for miRNA analysis | | |
| miR-330-5p | Stem-loop: | 5'-GTTGGCTCTGGTGCAGGGTCCGAGGTATTCGCACCAGAGCCAAC  GCCTAA-3' |
|  | Forward: | 5'-GTTTTCTCTGGGCCTGTGT-3' |
| miR-326-3p | Stem-loop: | 5'-GTTGGCTCTGGTGCAGGGTCCGAGGTATTCGCACCAGAGCCAAC  ACTGGA-3' |
|  | Forward: | 5'-TTGTCCTCTGGGCCCTTC-3' |
| miR-325-3p | Stem-loop: | 5'-GTTGGCTCTGGTGCAGGGTCCGAGGTATTCGCACCAGAGCCAAC  TTGATA-3' |
|  | Forward: | 5'-GGGTTTATTGAGCACCTCC-3' |
| U87 | Stem-loop: | 5'-GTTGGCTCTGGTGCAGGGTCCGAGGTATTCGCACCAGAGCCAAC  GCTCAG-3' |
|  | Forward: | 5'-GGTACAATGATGACTTATGTTTTTG-3' |
|  | Universal reverse: | 5'-GTGCAGGGTCCGAGGT-3' |

Drd1, dopamine receptor D1; Drd2, dopamine receptor D2; Slc6a3, solute carrier family 6 member 3; Th, tyrosine hydroxylase; Ubc, ubiquitin C; U87, small nucleolar RNA U87


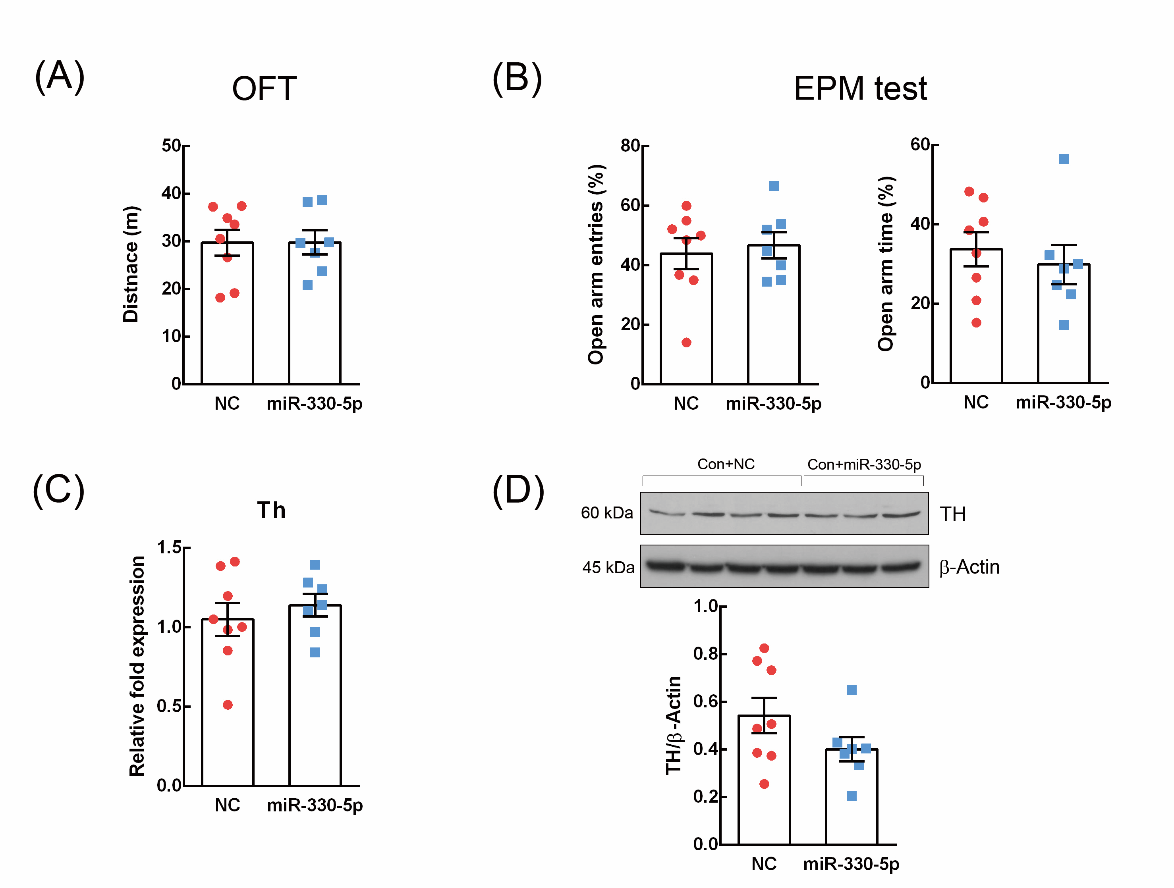


**Figure S2.** Effect of miR-330-5p on tyrosine hydroxylase (TH) expression and manic-like behaviors in control rats. TH mRNA and protein expressions were examined in the prefrontal cortex of control rats treated with negative control (NC) (n = 8) and miR-330-5p agomirs (n = 7) (D). Manic-like behaviors were measured using the open field test and elevated plus maze test in control rats treated with NC and miR-330-5p agomirs (n = 8 and 7 per group, respectively) (E). Expression levels of mRNA was normalized against those of Ubc. β-Actin expression was assessed as an internal control in western blotting. Results are shown as mean ± SEM. Differences between the groups were assessed by t-test. *p < 0.05 compared to NC agomir.


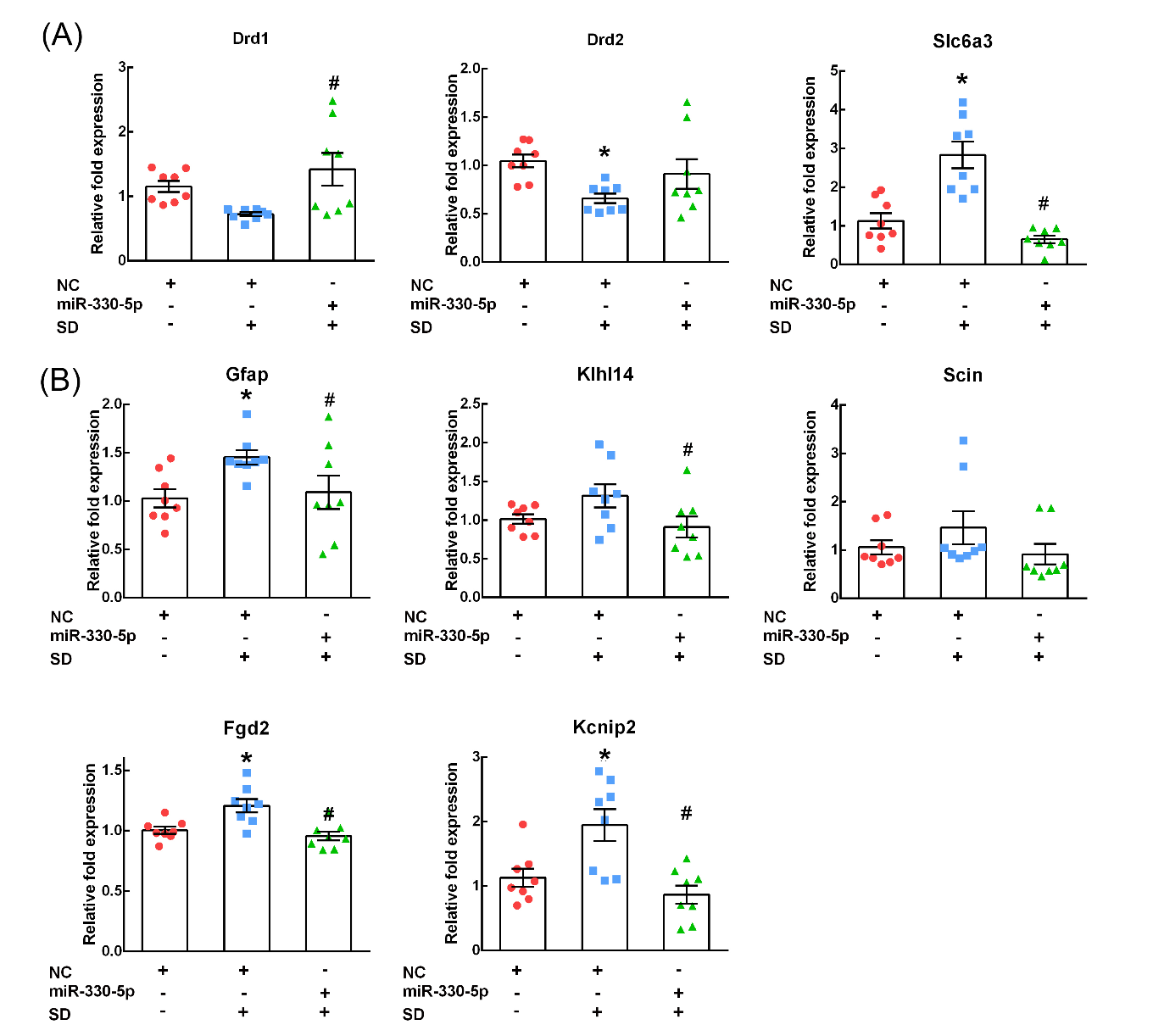


**Figure S3.** Expressions of dopamine synapse genes and target genes of miR-330-5p in the prefrontal cortex of miR-330-5p-treated SD rats. mRNA expressions of dopamine synapse genes (A) and miR-330-5p-target genes (B) were examined using quantitative real-time PCR (qRT-PCR) in the prefrontal cortex of SD rats injected with miR-330-5p agomir (n = 6 per group). miR-330-5p-target genes were selected among DEGs detected between control and SD groups.

**References**

1. Cousins DA, Butts K, Young AH. The role of dopamine in bipolar disorder. *Bipolar Disord.* 2009;11(8):787-806.

2. Gerner RH, Fairbanks L, Anderson GM, et al. CSF neurochemistry in depressed, manic, and schizophrenic patients compared with that of normal controls. *Am J Psychiatry.* 1984;141(12):1533-1540.

3. Manji HK, Quiroz JA, Payne JL, et al. The underlying neurobiology of bipolar disorder. *World Psychiatry.* 2003;2(3):136-146.

4. van Enkhuizen J, Geyer MA, Halberstadt AL, Zhuang X, Young JW. Dopamine depletion attenuates some behavioral abnormalities in a hyperdopaminergic mouse model of bipolar disorder. *J Affect Disord.* 2014;155:247-254.

5. Yu HS, Kim SH, Park HG, Kim YS, Ahn YM. Intracerebroventricular administration of ouabain, a Na/K-ATPase inhibitor, activates tyrosine hydroxylase through extracellular signal-regulated kinase in rat striatum. *Neurochem Int.* 2011;59(6):779-786.

6. O'Brien J, Hayder H, Zayed Y, Peng C. Overview of MicroRNA Biogenesis, Mechanisms of Actions, and Circulation. *Front Endocrinol (Lausanne).* 2018;9:402.

7. Karkhanis AN, Leach AC, Yorgason JT, et al. Chronic Social Isolation Stress during Peri-Adolescence Alters Presynaptic Dopamine Terminal Dynamics via Augmentation in Accumbal Dopamine Availability. *ACS Chem Neurosci.* 2019;10(4):2033-2044.

8. Riffo-Campos Á L, Riquelme I, Brebi-Mieville P. Tools for Sequence-Based miRNA Target Prediction: What to Choose? *Int J Mol Sci.* 2016;17(12).
