# Supplementary material for: Downregulation of microRNA‐330‐5p induces manic‐like behaviors in REM sleep‐deprived rats by enhancing tyrosine hydroxylase expression
Source: CNS Neurosci Ther. 2023 Feb 16;29(6):1525–36. doi: 10.1111/cns.14121 (PMC10173715; doi:10.1111/cns.14121)
Supplement: Supplementary file 1 — Appendix S1. [file CNS-29-1525-s001.zip › CNS_14121_supplementary_WB.pdf]

Full unedited gel/blot for Figure 2B

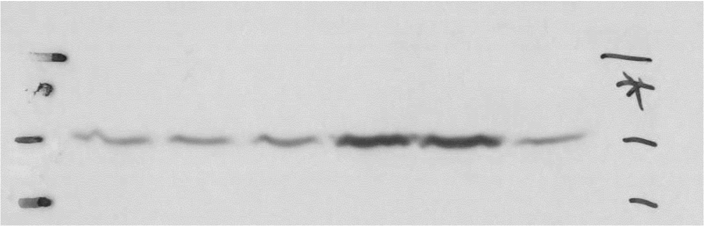

TH

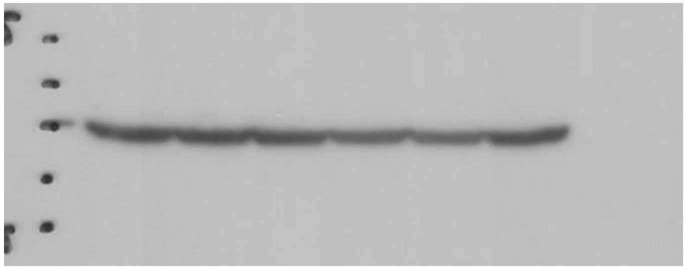

Beta-actin

Full unedited gel/blot for Figure 3

E

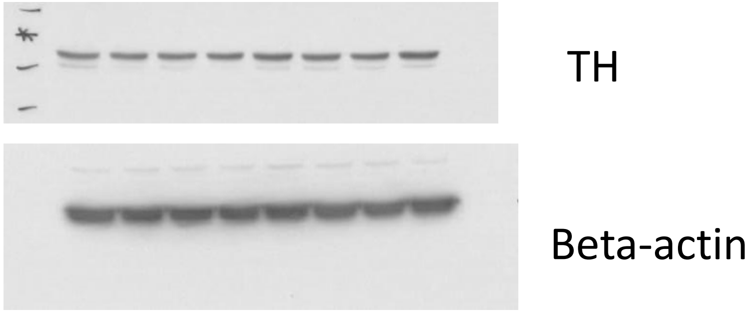

F

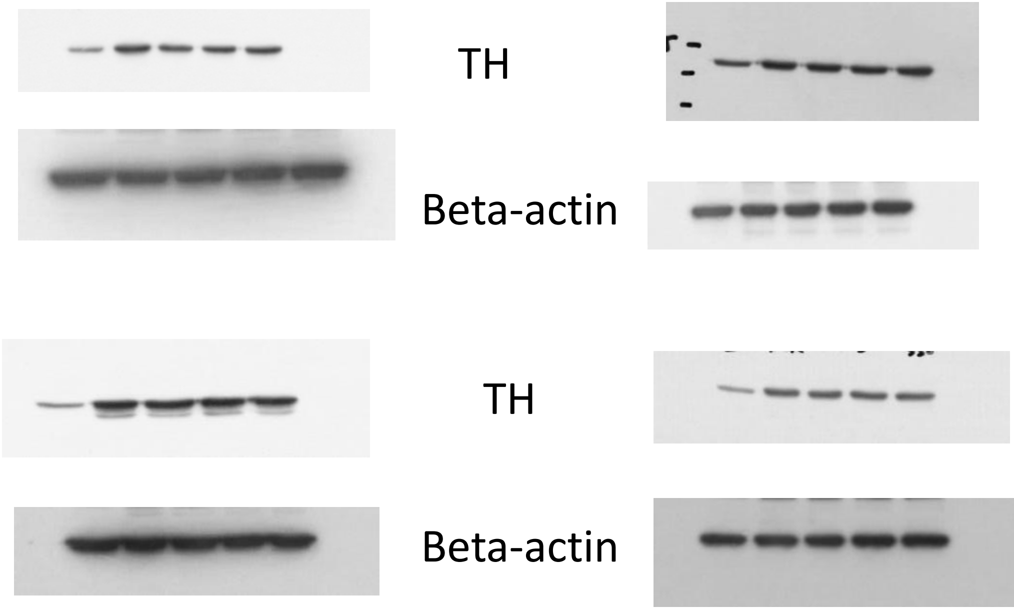

G

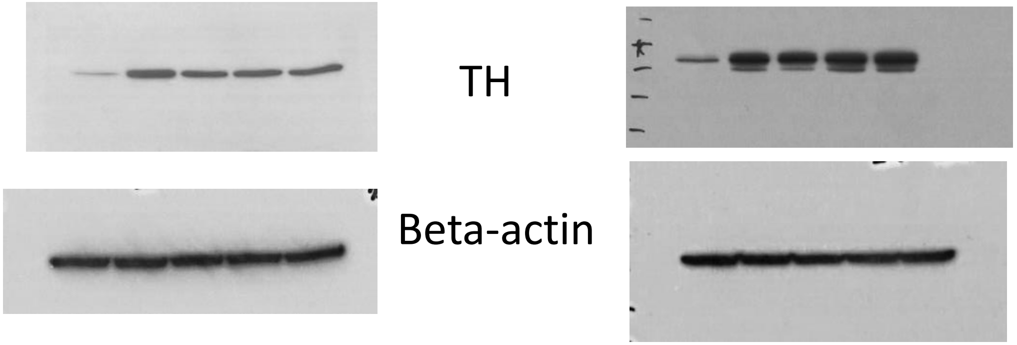

Full unedited gel/blot for Figure 5

A

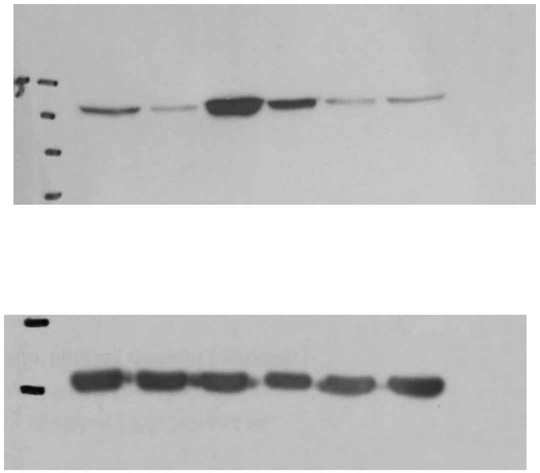

TH

Beta-actin

D

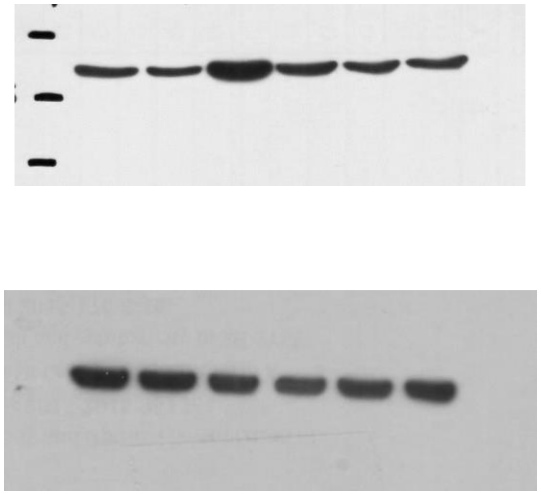

TH

Beta-actin

Full unedited gel/blot for supplementary Figure S2

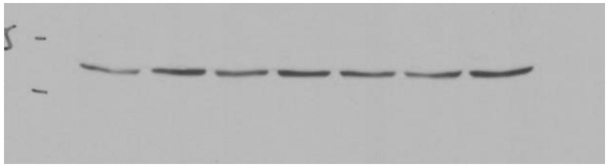

TH

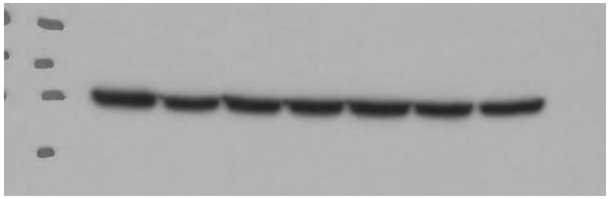

Beta-actin
